# Investigating the impact of regulatory B cells and regulatory B cell-related genes on bladder cancer progression and immunotherapeutic sensitivity

**DOI:** 10.1186/s13046-024-03017-8

**Published:** 2024-04-02

**Authors:** Jiawei Zhou, Ranran Zhou, Yuanchao Zhu, Shikai Deng, Bahaerguli Muhuitijiang, Chengyao Li, Xiaojun Shi, Ling Zhang, Wanlong Tan

**Affiliations:** 1 Department of Urology, Nanfang Hospital, Southern Medical University, Guangzhou, Guangdong Province 510080 China; 2 The First Clinical Medical College, Southern Medical University, Guangzhou, Guangdong 510080 China; 3 Department of Transfusion Medicine, School of Laboratory Medicine and Biotechnology, Southern Medical University, No. 1023-1063 Shatai South Road, Baiyun District, Guangzhou, Guangdong 510080 China

**Keywords:** Bladder cancer, Regulatory B cells, Gene signature, Prognosis, Immunotherapy

## Abstract

**Background:**

Regulatory B cells (Bregs), a specialized subset of B cells that modulate immune responses and maintain immune tolerance in malignant tumors, have not been extensively investigated in the context of bladder cancer (BLCA). This study aims to elucidate the roles of Bregs and Breg-related genes in BLCA.

**Methods:**

We assessed Breg infiltration levels in 34 pairs of BLCA and corresponding paracancerous tissues using immunohistochemical staining. We conducted transwell and wound healing assays to evaluate the impact of Bregs on the malignant phenotype of SW780 and T24 cells. Breg-related genes were identified through gene sets and transcriptional analysis. The TCGA-BLCA cohort served as the training set, while the IMvigor210 and 5 GEO cohorts were used as external validation sets. We employed LASSO regression and random forest for feature selection and developed a risk signature using Cox regression. Primary validation of the risk signature was performed through immunohistochemical staining and RT-qPCR experiments using the 34 local BLCA samples. Additionally, we employed transfection assays and flow cytometry to investigate Breg expansion ability and immunosuppressive functions.

**Results:**

Breg levels in BLCA tissues were significantly elevated compared to paracancerous tissues (*P* < 0.05) and positively correlated with tumor malignancy (*P* < 0.05). Co-incubation of SW780 and T24 cells with Bregs resulted in enhanced invasion and migration abilities (all *P* < 0.05). We identified 27 Breg-related genes, including CD96, OAS1, and CSH1, which were integrated into the risk signature. This signature demonstrated robust prognostic classification across the 6 cohorts (pooled HR = 2.25, 95% CI = 1.52–3.33). Moreover, the signature exhibited positive associations with advanced tumor stage (*P* < 0.001) and Breg infiltration ratios (*P* < 0.05) in the local samples. Furthermore, the signature successfully predicted immunotherapeutic sensitivity in three cohorts (all *P* < 0.05). Knockdown of CSH1 in B cells increased Breg phenotype and enhanced suppressive ability against CD8 + T cells (all *P* < 0.05).

**Conclusions:**

Bregs play a pro-tumor role in the development of BLCA. The Breg-related gene signature established in this study holds great potential as a valuable tool for evaluating prognosis and predicting immunotherapeutic response in BLCA patients.

**Supplementary Information:**

The online version contains supplementary material available at 10.1186/s13046-024-03017-8.

## Introduction

Bladder cancer (BLCA) is ranked as the 10th most prevalent cancer globally, with approximately 573,000 new cases and 213,000 deaths [1, 2]. According to reports from the World Health Organization, the incidence of BLCA is projected to double by 2040 [3]. BLCA can be categorized into two distinct subtypes based on the depth of tumor invasion in the bladder wall: non-muscle-invasive BLCA (NMIBC) and muscle-invasive BLCA (MIBC) [4]. While the prognosis for NMIBC patients is generally favorable, around 15–20% of NMIBC cases progress to MIBC, resulting in significantly worse clinical outcomes. Despite significant advancements in BLCA treatment in recent years, including the introduction of neoadjuvant chemotherapy and targeted therapy, a considerable number of BLCA patients do not benefit from these treatment regimens due to the extensive morphological and molecular heterogeneity of the disease [5]. BLCA exhibits a high mutational burden, ranking third only to melanoma and lung cancers, thereby underscoring the potential of targeting the immune system as a promising therapeutic strategy against BLCA [6]. The recent approval of immune checkpoint inhibitors, particularly the anti-PD1/PDL1 reagent, has revolutionized BLCA treatment by demonstrating prolonged response duration and clinical benefits in BLCA cases [7]. However, the IMvigor210 clinical trial revealed that only approximately 20% of BLCA patients exhibited a response to atezolizumab (an anti-PD1 antibody) treatment [8]. Therefore, the imminent challenges encompass identifying novel therapeutic targets for new drug discovery and developing reliable biomarkers to enable personalized prognosis assessment, thereby advancing the field of precision medicine and efficacy prediction and promoting the prognoses of BLCA patients.

Regulatory B cells (Bregs) represent a specialized subset of B cells renowned for their immunosuppressive properties, which are crucial in maintaining immune tolerance and regulating inflammation [9, 10]. Initially identified in the context of autoimmunity, Bregs have now been acknowledged for their significant roles in infection, allergy, and transplant tolerance [11]. In the realm of tumor biology, Bregs have been found to impede anti-tumor immune responses, thereby promoting tumorigenesis. Furthermore, the infiltration of Bregs has been associated with unfavorable prognoses in various cancers [12–14]. These effects are achieved through diverse mechanisms, including the secretion of immunosuppressive cytokines such as IL10, IL35, and TGFβ, which suppress the activation and function of effector T cells [15]. Bregs also interact directly with other immune cells, such as dendritic cells, macrophages, NK cells, and regulatory T cells (Tregs), to foster an immunosuppressive milieu [16, 17]. In the context of BLCA, a previous study investigated the expression of IL10 in B cells and its impact on BLCA subjects, revealing a negative influence and suggesting a nonnegligible role of Bregs in the pathogenesis of BLCA [18]. However, the functions of Bregs on BLCA cells and the underlying biological mechanisms remain unexplored. Moreover, a comprehensive analysis of the prognostic value of Breg-related genes in cancer, especially BLCA, has yet to be conducted.

This study is divided into two main parts. Firstly, we collected 34 pairs of BLCA tissues and their corresponding paracancerous tissues from a local hospital. To quantify the infiltration levels of Bregs, we employed dual immunohistochemical staining using CD19 and IL10 as markers. B cells were isolated from normal human peripheral blood mononuclear cells (PBMCs) and educated by BLCA cells SW780 and T24 to generate Bregs. The co-incubation of SW780 and T24 cells with Bregs allowed us to assess the malignant phenotypes of BLCA cells using transwell invasion and wound healing assays.

Secondly, we constructed a Breg-related gene signature in BLCA. Breg-related genes were selected by curating immunosuppressive gene sets, Breg cell marker genes, and genes with differential expression between IL10- and IL10 + B cells. For training, we handpicked a cohort of 399 BLCA subjects from The Cancer Genome Atlas (TCGA). We utilized the IMvigor210 cohort and 5 BLCA datasets obtained from the Gene Expression Omnibus (GEO) to ensure robust external validation. To identify significant Breg-related genes associated with Overall Survival (OS), we employed the Least Absolute Shrinkage and Selection Operator (LASSO) regression technique in conjunction with the random forest algorithm. The resulting risk multiple-gene signature was constructed using multivariate Cox regression. Additionally, comprehensive pan-cancer analyses were conducted to investigate the prognostic value of this risk signature in other cancers. Finally, transfection assays were performed in B cells to gain further insights into the influence of the genes within the signature on Breg expansion and immunosuppressive functions to CD8 + T cells.

## Materials and methods

### Detecting the effect of bregs on the development of BLCA

Between 2021 and 2022, 34 pairs of BLCA tissues and their corresponding paracancerous tissues were collected from the Nanfang Hospital of Southern Medical University. Dual immunohistochemical staining was performed using CD19 (proteintech, China) and IL10 (proteintech, China) markers to assess the levels of Bregs. Clinicopathological features, including tumor-node-metastasis (TNM) stages, age, and gender, were also recorded.

In parallel, B cells and CD8 + T cells were isolated from the PBMCs of 5 healthy human subjects using magnetic beads. The B cells were then co-incubated with T24 and SW780 cells to generate Bregs [17, 19]. Flow cytometry was employed to measure the expansion ability of Bregs. Subsequently, these educated B cells were co-cultured with T24 and SW780 cells, and the invasion and migration capabilities of T24 and SW780 cells were assessed using transwell and wound healing assays.

### Breg-related gene signature construction and validation

Breg-related genes were collected from various sources, including immunosuppressive gene sets from Gene Ontology (GO, https://geneontology.org/), co-expression genes of IL10, TGFβ, and IL35 from GeneMANIA (http://genemania.org/), Breg cell marker genes from CellMarker (http://bio-bigdata.hrbmu.edu.cn/CellMarker/), and differentially-expressed genes (DEGs) between IL10- and IL10 + B cells from GEO (GSE50895, https://ncbi.nlm.nih.gov/geo/) [19]. The robustness of these Breg-related genes was verified through Gene Set Enrichment Analysis (GSEA) of 326 B cells from scRNAseqDB (https://bioinfo.uth.edu/scrnaseqdb/) and Western Blot analysis of IL10- and IL10 + B cells.

Transcriptional profiles, clinicopathological parameters, and follow-up information of subjects across 33 different cancers, including BLCA, were downloaded from the UCSC Xena website (http://xena.ucsc.edu/). The TCGA-BLCA cohort was used as the training cohort, while 5 independent BLCA cohorts (GSE13507 [20], GSE31684 [21], GSE32894 [22], GSE5287 [23], and IMvigor210) were selected for external validation (Table S1). Additionally, a cohort of patients with advanced melanoma before nivolumab treatment was analyzed to verify the predictive ability of the risk model to immunotherapeutic sensitivity [24].

LASSO regression with 10-fold cross-validation and random forest algorithms were employed to identify Breg-related genes with significant prognostic value. These genes were included in the multivariate Cox regression model to calculate the risk score, known as the Breg-related score (BREGRS), for each subject, as follows: $\text{B}\text{R}\text{E}\text{G}\text{R}\text{S}={\sum }_{i=1}^{n}{{Coeff}_{i}*Exp\left(Gene\right)}_{i}$, where “Coeff” represented the coefficients of the variables in the Cox regression model and “Exp” meant the mRNA expression levels of the genes. Meta-analyses were conducted to combine hazard ratios (HRs) and better understand the prognostic value of BREGRS.

The association between BREGRS and BLCA malignant stages was verified in 34 local clinical samples using immunohistochemical staining and real-time quantitative PCR (RT-qPCR). Transient transfection assays on B cells from healthy human PBMCs were performed using small-interference RNA (siRNA). The primer and siRNA sequences are shown in Tables S2 and S3, respectively. The effects of siRNA treatment on B cells were then evaluated through co-incubation with CD8 + T cells, and cytokine (IFNγ and TNFα) production assays of CD8 + T cells were measured using flow cytometry analysis.

### Analysis of immunogenetic profiles

The ESTIMATE algorithm was used to calculate the abundance of stromal and immune components in the tumor microenvironment. The TCGA-BLCA subjects’ immune subtype classification was obtained from a previous study, with five main immune subtypes: IFNγ Dominant, Inflammatory, Lymphocyte Depleted, TGFβ Dominant, and Wound Healing [25]. The infiltration levels of various immune cells, such as T cells, B cells, NK cells, and Macrophages, were evaluated using the CIBERSORT-ABS and XCELL algorithms. Gene sets from 7 immune-related pathways, including TNFα signaling via NF-kB, IL2 STAT5 signaling, IL6 JAK STAT3 signaling, inflammatory response, IFNα response, IFNγ response, and complement, were collected from the HALLMARK gene sets downloaded from the Molecular Signatures Database (MSigDB, https://www.gsea-msigdb.org/gsea/msigdb/). We also analyzed the expression difference of the human leukocyte antigen (HLA) gene family, responsible for presenting foreign antigens to immune cells, and immune checkpoint genes, which regulate T cell activation and immune surveillance activity. The Tumor Immune Dysfunction and Exclusion (TIDE) algorithm (http://tide.dfci.harvard.edu) was used to evaluate the response to immune checkpoint inhibitor therapy in the TCGA-BLCA cases.

### Statistical analysis

The statistical analyses for the entire study were conducted using R software (version 4.2.0). Differences in the experimental data were assessed using paired Student’s t-test or Welch’s corrected t-test. For the bioinformatical analysis, comparisons were made using the Wilcoxon signed-rank test or the Kruskal-Wallis test. Categorical variables were compared using Pearson’s chi-square or Fisher’s exact tests. OS differences throughout the follow-up period were analyzed using Kaplan-Meier with a log-rank test or two-stage hazard rate comparison. A more detailed methodology can be found in Supplementary File 2.

## Results

### Bregs promote the tumorigenesis of BLCA

**Fig. 1:**
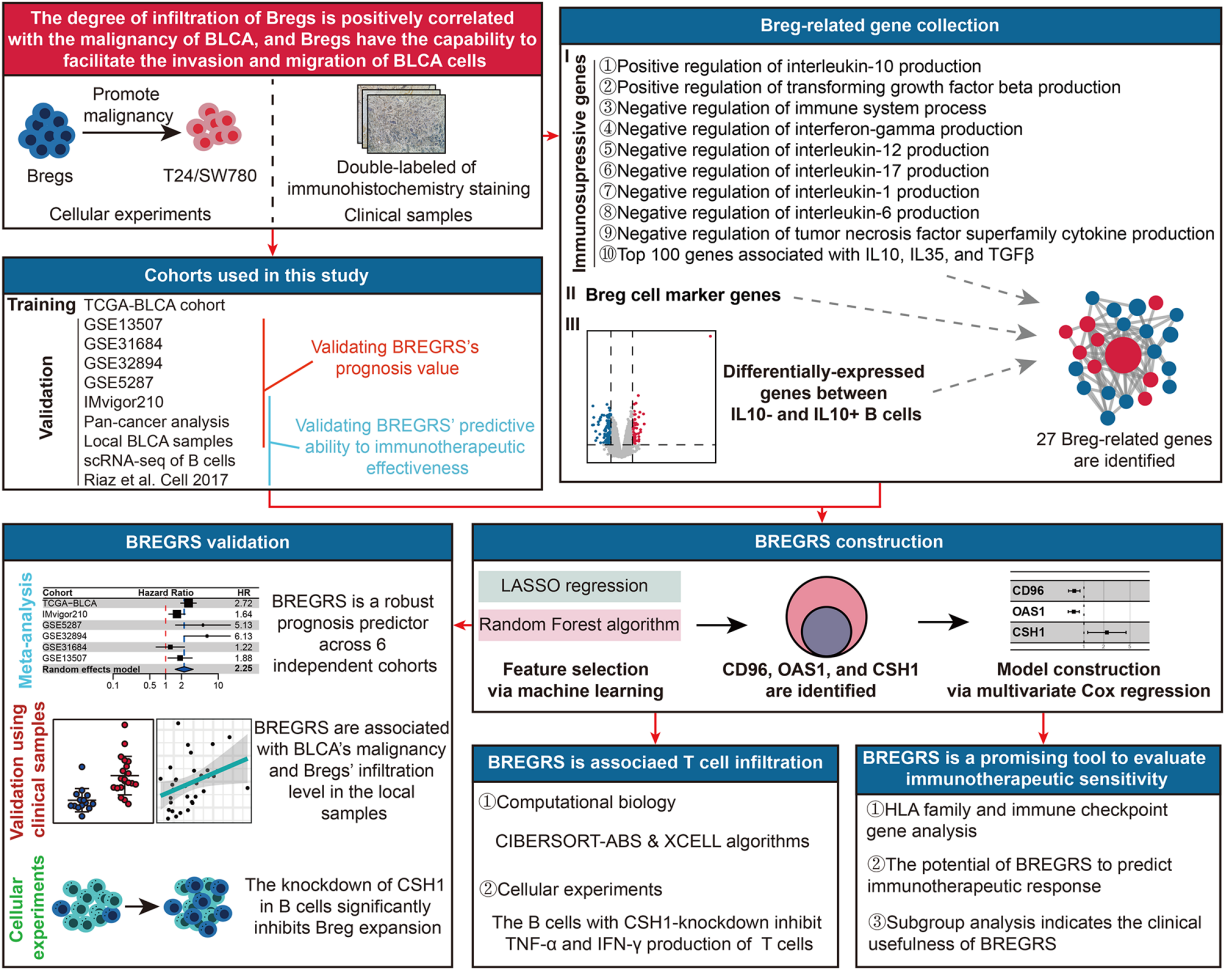
Overview of the study design, which was divided into two main parts. In the first part, we collected 34 pairs of BLCA tissues and their corresponding paracancerous tissues from a local hospital. The infiltration levels of Bregs in these tissues were quantified using dual immunohistochemical staining. Additionally, we examined the malignant phenotypes of BLCA cells T24 and SW780 when co-incubated with Bregs. In the second part, we gathered Breg-related genes from various sources and developed a Breg-related signature. This signature was created using LASSO regression, random forest analysis, and multivariate Cox regression based on the TCGA-BLCA cohort. To validate the prognostic value of the risk signature, we tested it on six independent BLCA cohorts and local BLCA samples. Furthermore, we assessed the predictive ability of the signature for immunotherapeutic sensitivity using two cohorts that received anti-PD1/PDL1 treatment and the TIDE algorithm. Finally, we conducted cellular functional experiments to investigate the regulatory ability of CSH1, a gene involved in the risk signature, in the expansion of Bregs. *BLCA, bladder cancer; Bregs, regulatory B cells; LASSO, Least Absolute Shrinkage and Selection Operator; TCGA, The Cancer Genome Atlas; TIDE, Tumor Immune Dysfunction and Exclusion*

**Fig. 2:**
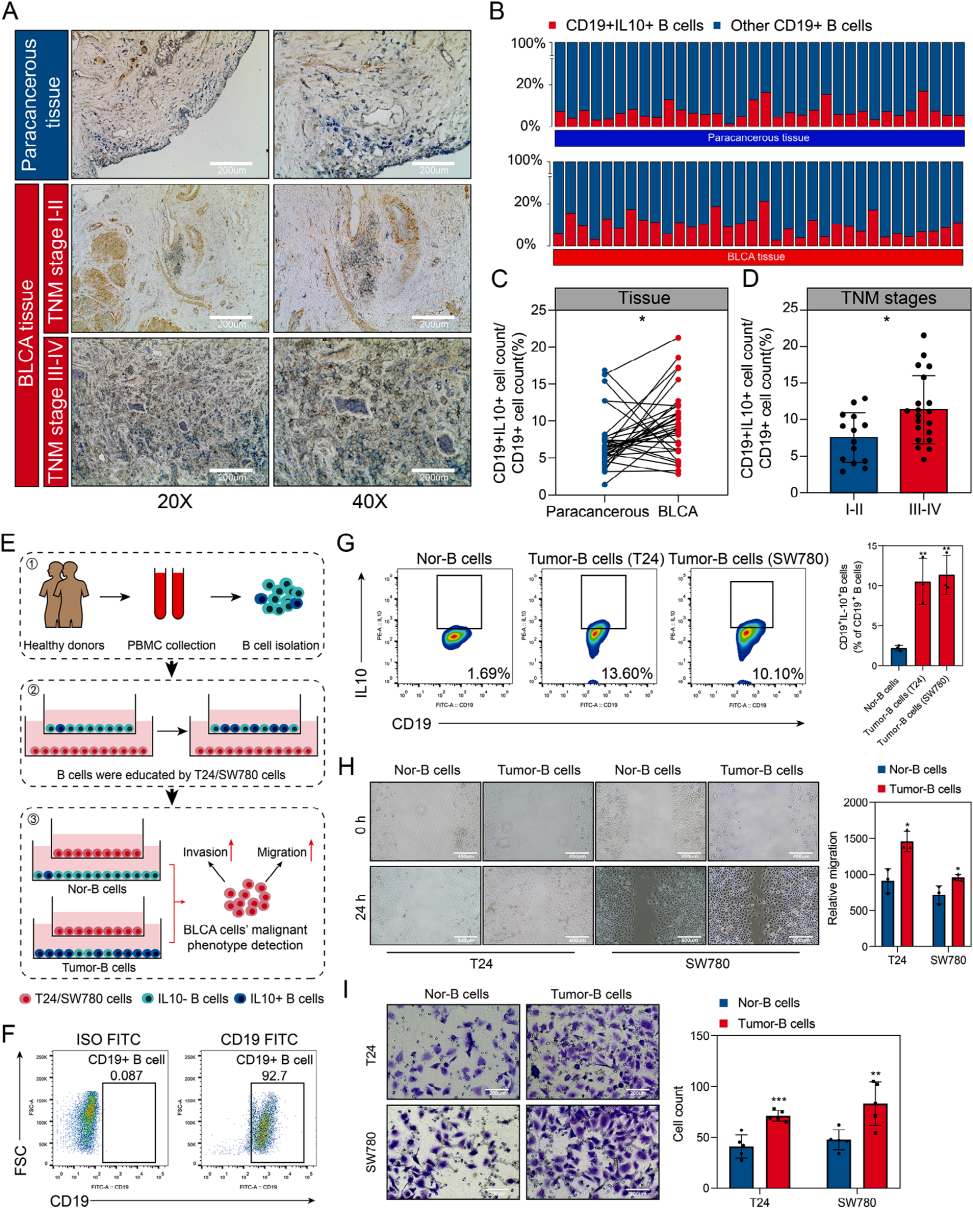
Bregs promote the development of BLCA. (**A**) The infiltration levels of Bregs were quantified using dual immunohistochemical staining of CD19 and IL10. CD19 was visualized with blue staining, while IL10 was represented by brown staining. (**B**) Infiltration levels of Bregs in clinical samples obtained from a local hospital. (**C**) Higher infiltration levels of Bregs were observed in BLCA tissues compared to paracancerous tissues. (**D**) Infiltration levels of Bregs were associated with advanced TNM stages in BLCA samples. (**E**) Experimental design to demonstrate the pro-tumor functions of Bregs. (**F**) Purity of B cells isolated from PBMC using magnetic beads was assessed through flow cytometry analysis. (**G**) B cells educated by SW780 and T24 cells exhibited enhanced Breg features. (**H, I**) T24 and SW780 cells co-incubated with educated B cells displayed increased migration (**H**) and invasion (**I**) abilities. PBMC, peripheral blood mononuclear cells; *P < 0.05; **P < 0.01; ***P < 0.001

Figure 1 illustrates the workflow of our study. Initially, we utilized clinical samples from our hospital and conducted in vitro experiments to demonstrate the pro-tumor functions of Bregs in BLCA. We performed dual immunohistochemical staining of CD19 and IL10 on 34 BLCA and corresponding paracancerous tissues (Fig. 2A) to quantify the levels of Bregs infiltration (Fig. 2B, Table S4). BLCA tissues exhibited a higher infiltration ratio of Bregs compared to paracancerous tissue (*P* < 0.05, Fig. 2C). Moreover, the proportion of Bregs infiltration was found to be associated with advanced TNM classification (*P* < 0.05, Fig. 2D). We also examined the association of Bregs abundance with other clinical features such as age and gender (Fig. S1).

B cells were isolated from PBMCs obtained from healthy human subjects using magnetic beads and then exposed to BLCA cells T24 and SW780 to generate Bregs (Fig. 2E). The efficacy of the magnetic beads was confirmed through flow cytometry analysis (Fig. 2F). Co-incubation with T24 or SW780 resulted in higher levels of Bregs compared to the control group (both *P* < 0.01, Fig. 2G). Subsequently, these B cells were co-incubated with T24 and SW780 cells (Fig. 2E), leading to increased migration (both *P* < 0.05, Fig. 2H) and invasion (both *P* < 0.01, Fig. 2I) characteristics in these BLCA cells. Moreover, in order to gain a better understanding of the pro-tumor characteristics of Bregs, we conducted a comprehensive analysis using the GeneCards database and identified the Top 20 genes that were strongly associated with BLCA (Table S5). We then assessed the expression levels of these genes in T24 (Fig. S2A) and SW780 (Fig. S2B) cells that were co-incubated with Bregs. Significantly, we observed differential expression of several well-established cancer promoter and suppressor genes, including TP53 (both *P* < 0.001), PTEN (both *P* < 0.001), EGFR (both *P* < 0.001), PIK3CA (both *P* < 0.001), and KRAS (both *P* < 0.01), in these cells.

### Collection of breg-related genes

**Fig. 3:**
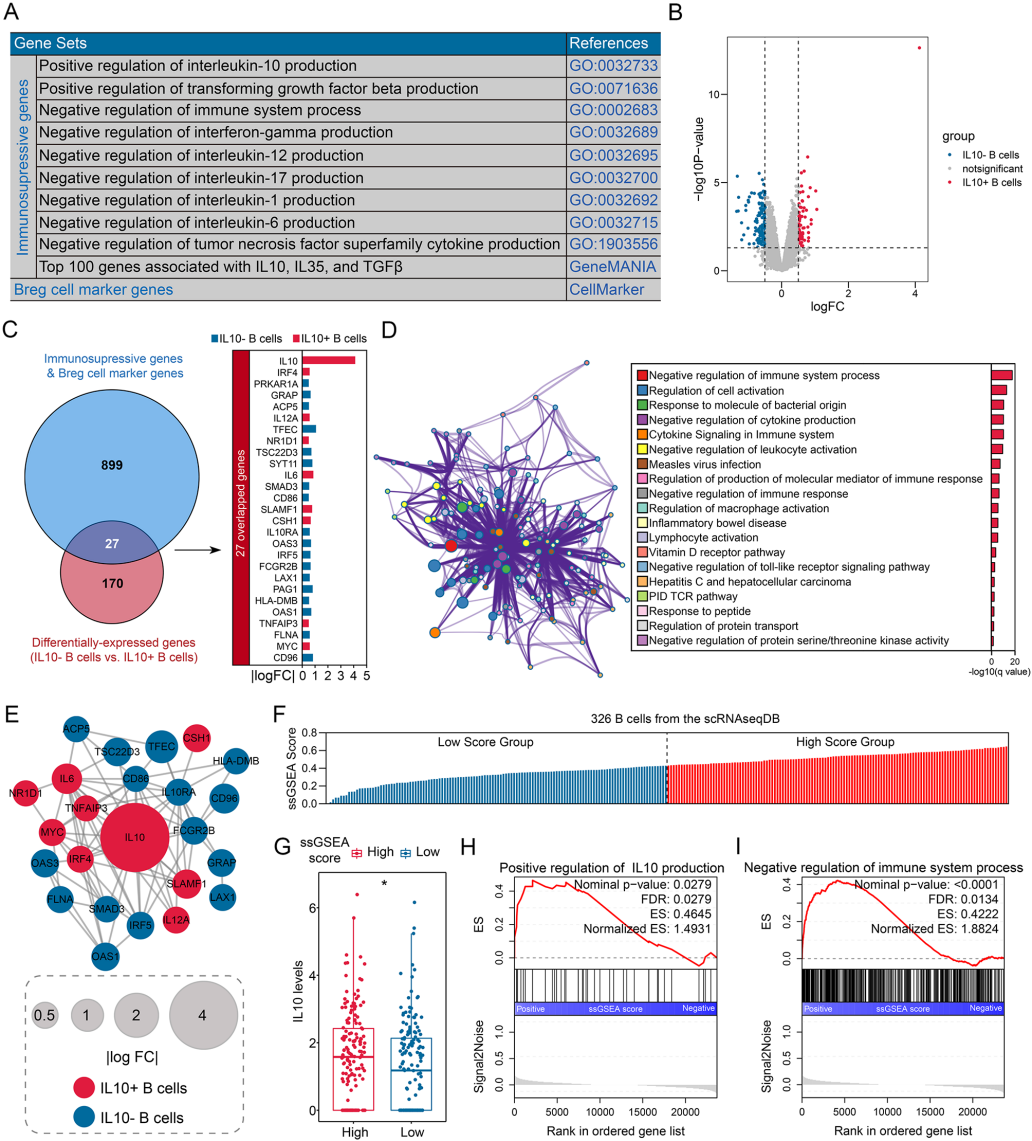
Identification of Breg-related genes. (**A**) We compiled immunosuppressive gene sets and Breg cell marker genes. (**B**) Differentially-expressed genes between IL10- and IL10 + B cells were analyzed. (**C**) A total of 27 genes were identified as Breg-related genes, which were found to overlap. (**D**) Functional enrichment analysis was conducted to understand the biological roles of the 27 genes. (**E**) A PPI network was constructed to reveal the internal interactions among the 27 genes. (**F**) The expressions of the 27 Breg-related genes were used to compute the ssGSEA scores for 326 B cells extracted from the scRNAseqDB. (**G**) The levels of IL10 were compared between the high- and low-ssGSEA score subgroups. (**H**) Positive associations were observed between the ssGSEA score and the regulation of IL10 production. (**I**) Negative associations were found between the ssGSEA score and the regulation of immune system processes. ssGSEA, single-sample Gene Set Enrichment Analysis; PPI, protein-protein interaction; *P < 0.05; **P < 0.01; ***P < 0.001

Breg-related genes were defined by combining immunosuppressive gene sets, IL10, TGFβ, and IL35 co-expression genes, reported Breg marker genes, and DEGs between IL10 + and IL10- B cells. From the GO database, we obtained a total of 592 immunosuppressive genes (Table S6). Additionally, using the GeneMANIA database, we identified 374 co-expression genes of IL10, TGFβ, and IL35 (Fig. S3). Breg cell marker genes were determined through the CellMarker website (Table S6). Figure 3A provides a concise summary of these gene sets. Subsequently, we analyzed the DEGs between IL10- (n = 5) and IL10+ (*n* = 5) B cells, resulting in the identification of 197 DEGs, including 67 up-regulated and 130 down-regulated genes (Fig. 3B, Table S7). Ultimately, we determined 27 Breg-related genes (Fig. 3C). Functional enrichment analysis revealed that these genes are primarily involved in immune-related pathways, particularly in the negative regulation of immunity (Fig. 3D, Table S8). The protein-protein interaction (PPI) network (Fig. 3E) and co-expression analyses (Fig. S4) further confirmed the close association of these genes.

Additionally, to validate the association of the 27 genes with Bregs’ characteristics, we performed single-sample GSEA (ssGSEA) on 326 B cell samples from the scRNAseqDB. These samples were divided into low- and high-score subgroups based on the median ssGSEA score (Fig. 3F). Notably, the high-score subgroup exhibited significantly higher levels of IL10 (*P* < 0.05, Fig. 3G). Moreover, the scores were positively correlated with the regulation of IL10 production (False Discovery Rate [FDR] < 0.05, Normalized Enrichment Score [NES] = 1.49, Fig. 3H) and negatively correlated with the regulation of immune system processes (FDR < 0.05, NES = 1.88, Fig. 3I).

### Establishment and primary validation of BREGRS

**Fig. 4:**
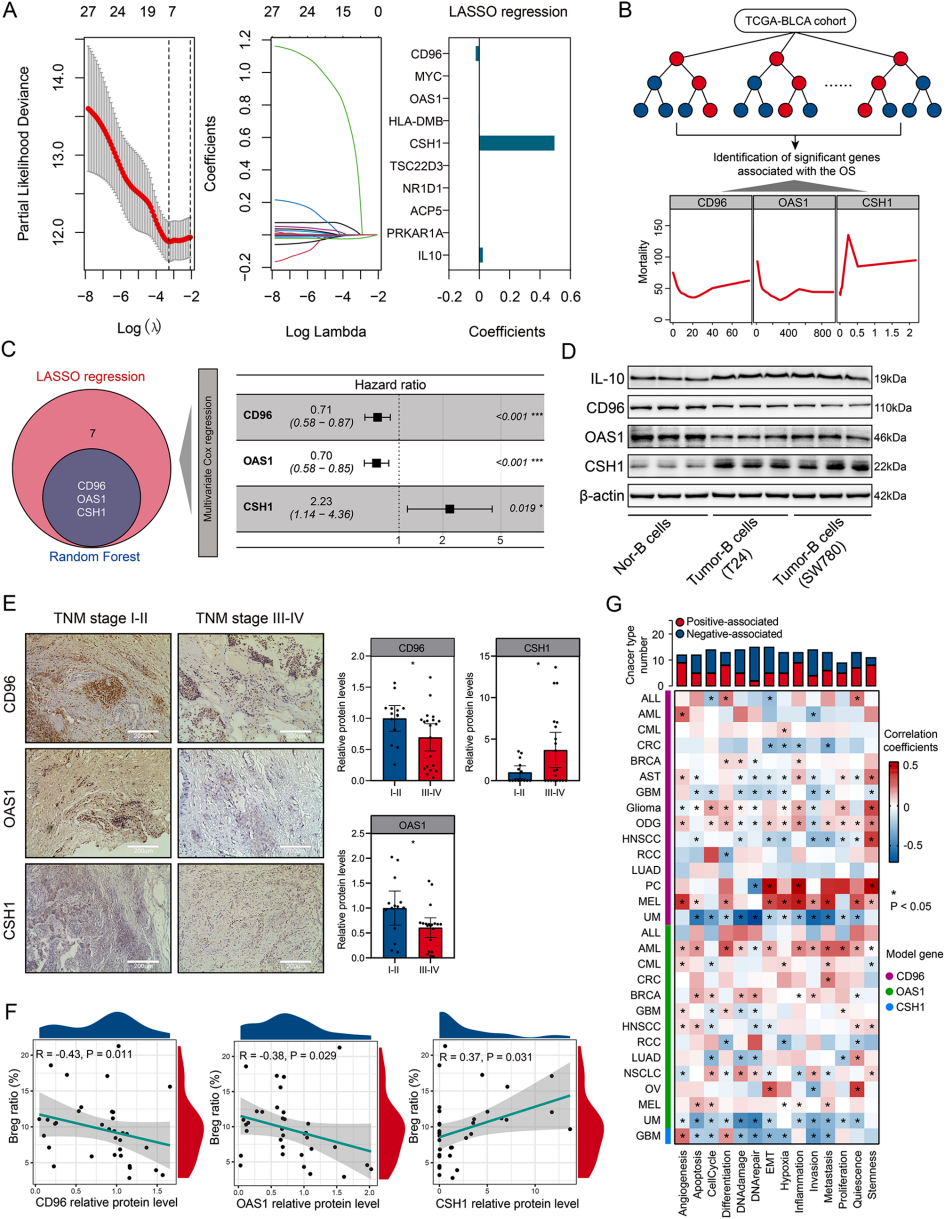
Construction of BREGRS. (**A**) LASSO regression analysis identified 10 out of the 27 Breg-related genes as significant predictors for OS. (**B**) Random forest analysis identified 3 out of the 27 Breg-related genes as significant prognostic factors. (**C**) CD96, OAS1, and CSH1 were determined as significant genes by both LASSO and random forest analyses, and were included in the multivariate Cox regression model. (**D**) Protein expression levels of CD96, OAS1, and CSH1 were evaluated in B cells with and without BLCA cells’ education. (**E**) Immunohistochemical staining of CD96, OAS1, and CSH1 in local BLCA samples revealed their association with BLCA TNM stages. (**F**) Correlation analysis was performed to assess the relationship between protein expressions of CD96, OAS1, and CSH1 and infiltration levels of Bregs in the local cohort. (**G**) Investigation of the association of CD96, OAS1, and CSH1 with multiple malignant phenotypes at a single-cell-based pan-cancer level. BREGRS, Breg-related score; LASSO, Least Absolute Shrinkage and Selection Operator; *P < 0.05; **P < 0.01; ***P < 0.001

To explore the prognostic significance of Breg-related genes in BLCA cases, we employed LASSO regression and random forest for feature selection. LASSO regression identified 10 genes (Fig. 4A, Table S9), three of which were also identified by the random forest analysis (Fig. 4B). Ultimately, CD96, OAS1, and CSH1 were co-determined and included in the multivariate Cox regression model (Fig. 4C).

A series of experiments were conducted to verify our findings. Compared to control B cells, B cells educated by BLCA cells exhibited decreased protein expression of CD96 and OAS1 and increased protein levels of CSH1 (all *P* < 0.05, Fig. 4D, Fig. S5). Immunohistochemical staining of 34 BLCA samples collected from the local hospital revealed that cases with advanced TNM stages had lower levels of CD96 and OAS1, and higher levels of CSH1 (all *P* < 0.05, Fig. 4E). Notably, the protein levels of CD96 (*R* = -0.43, *P* < 0.05), OAS1 (*R* = -0.38, *P* < 0.05), and CSH1 (*R* = 0.37, *P* < 0.05) were significantly associated with the infiltration proportion of Bregs in the local samples (Fig. 4F). Single-cell pan-cancer analysis conducted on the CancerSEA database (http://biocc.hrbmu.edu.cn/CancerSEA/) demonstrated a clear correlation between CD96, OAS1, CSH1, and multiple malignant phenotypes in different cancers (Fig. 4G). Overall, our study suggests that CD96, OAS1, and CSH1 are strongly associated with Bregs and serve as reliable prognostic biomarkers in BLCA.

### BREGRS is a reliable prognostic predictor in BCLA

**Fig. 5:**
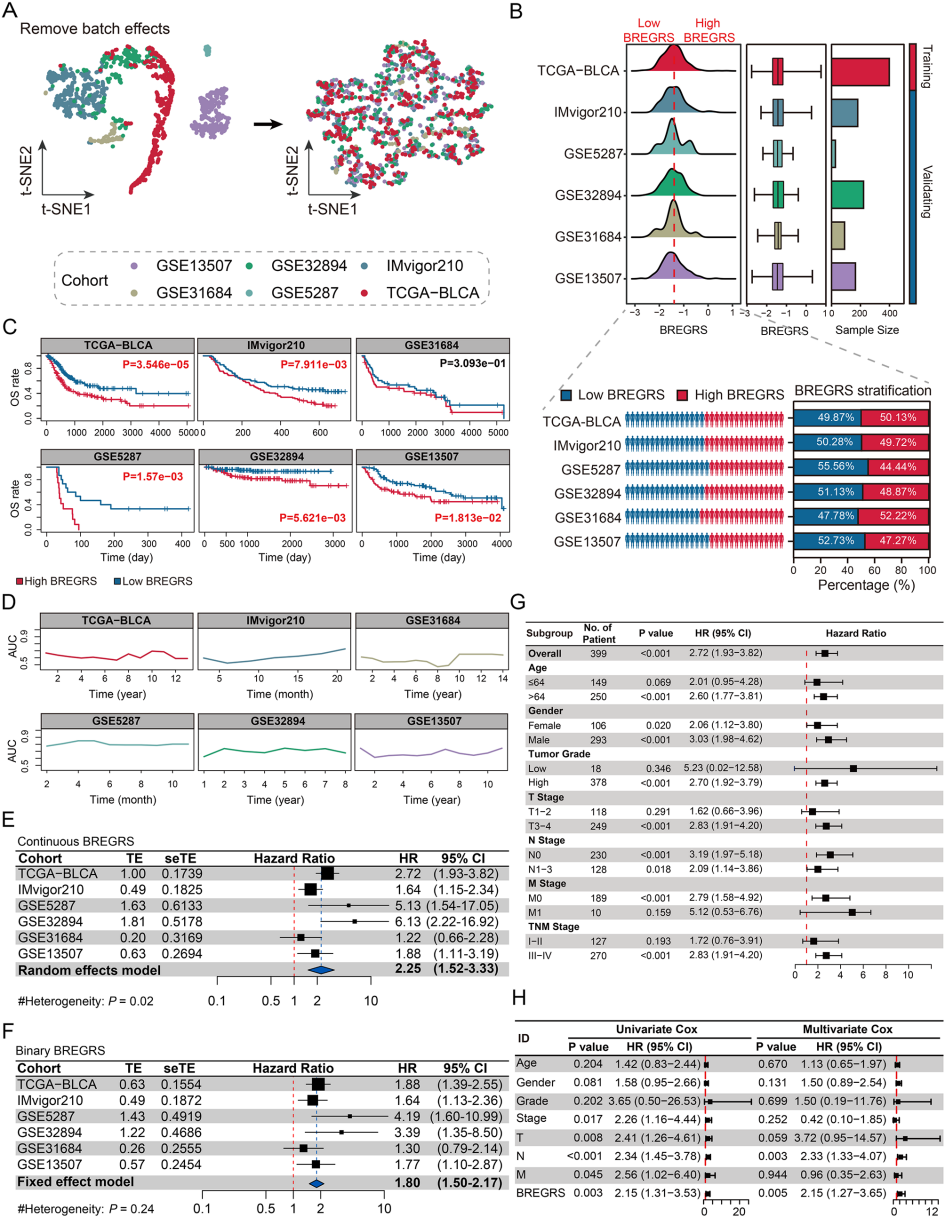
BREGRS serves as a reliable prognosis predictor in BLCA. (**A**) The t-SNE algorithm was used to assess the effectiveness of removing batch effects in the BLCA cohorts. (**B**) BREGRS was calculated for each subject in the BLCA cohorts. Based on the median BREGRS level in the training cohort (TCGA-BLCA), all BLCA cases were divided into low- and high-BREGRS subgroups. (**C, D**) Kaplan-Meier survival (**C**) and ROC (**D**) analyses indicated the prognosis value of BREGRS in these cohorts. (**E, F**) Meta-analyses were conducted to calculate the pooled HRs of BREGRS in these cohorts using the continuous (**E**) and binary (**F**) BREGRS. (**G**) Subgroup analyses of BREGRS were performed to explore its prognostic value in different patient subgroups. (**H**) Univariate and multivariate Cox regression analyses were conducted to assess the independent prognostic value of BREGRS. t-SNE, t-distributed Stochastic Neighbor Embedding; ROC, receiver operating characteristic; HR, Hazard Ratio

The predictive ability of BREGRS for OS in BLCA subjects was assessed in multiple cohorts, including TCGA-BLCA (*n* = 399), IMvigor210 (*n* = 181), GSE5287 (*n* = 27), GSE13507 (*n* = 165), GSE31684 (*n* = 90), and GSE32894 (*n* = 221). The baseline clinicopathological features of these cohorts can be found in Table S10. We used the “sva” R package to eliminate the batch effect (Fig. 5A), and BREGRS was calculated for the cases in each cohort (Fig. 5B). Using the same cut-off value (-1.427) as the median BREGRS in the training cohort, all cases were divided into high- and low-BREGRS subgroups (Fig. 5B). Dimension-reduction algorithms indicated distinct genomic features between the high- and low-BREGRS subgroups (Fig. S6). The OS difference was then evaluated in the training (*P* < 0.001), IMvigor210 (*P* < 0.01), GSE31684 (*P* > 0.05), GSE5287 (*P* < 0.01), GSE32894 (*P* < 0.01), and GSE13507 (*P* < 0.05) cohorts (Fig. 5C). Time-dependent Receiver Operating Characteristic (ROC) analyses demonstrated that the Areas Under Curves (AUCs) at different time points were consistently above 0.5, indicating the robustness of BREGRS (Fig. 5D). Since no significant result was observed in the GSE5287 cohort, possibly due to the limited sample size (GSE5287, *n* = 27), meta-analyses were conducted to combine the effect values. The meta-analyses confirmed that BREGRS was a reliable prognostic indicator across six independent cohorts, whether using continuous BREGRS (pooled HR = 2.25, 95% Confidence Interval [CI] = 1.52–3.33, Fig. 5E) or binary BREGRS (pooled HR = 1.80, 95% CI = 1.50–2.17, Fig. 5F). Further analysis revealed that BREGRS was particularly applicable to BLCA cases with age > 64 (HR = 2.60, 95% CI = 1.77–3.81), male gender (HR = 3.03, 95% CI = 1.98–4.62), high tumor grade (HR = 2.70, 95% CI = 1.92–3.79), pathological T3-4 stages (HR = 2.83, 95% CI = 1.91–4.20), pathological N1-3 stages (HR = 2.09, 95% CI = 1.14–3.86), M0 stage (HR = 2.79, 95% CI = 1.58–4.92), or TNM stage III-IV (HR = 2.83, 95% CI = 1.91–4.20) (all *P* < 0.001, Fig. 5G). Additionally, after transforming the clinicopathological parameters into binary variables, BREGRS served as an independent prognostic predictor in both univariate and multivariate Cox regression analyses (both *P* < 0.01, Fig. 5H).

We obtained the established prognosis signatures linked to B cell profiles in BLCA from published literature (Table S11) [26–29]. Risk scores for the TCGA-BLCA subjects were computed using the provided formula (Table S12). Univariate Cox regression analysis revealed that BREGRS exhibited the highest HR with *P* < 0.05 (HR = 2.72, 95% CI = 1.93–3.82, Fig. S7), indicating the superior prognostic prediction capability of BREGRS in BLCA.

### Analysis of the prognosis value of BREGRS at a pan-cancer level

Given that constructing a Breg-related prognosis signature in malignant cancers is a novel endeavor, we conducted a comprehensive pan-cancer analysis to elucidate the prognostic value of BREGRS in 32 other cancers derived from the TCGA dataset. Notably, CD96 exhibited significant predictive ability for OS in 9 cancers (all *P* < 0.05, Table S13), CSH1 demonstrated significance in 3 cancers (all *P* < 0.05, Table S14), and OAS1 emerged as a significant predictor in 10 cancers (all *P* < 0.05, Table S15). Employing the aforementioned established formula, we computed the BREGRS for each subject (Fig. S8). Remarkably, BREGRS emerged as a significant prognostic factor in 18 cancer types (all *P* < 0.05, Table S16) and was associated with unfavorable OS in 9 cancers (all *P* < 0.05, Fig. S9). The considerable immune heterogeneity observed across different cancer types may account for this divergence of BREGRS, yet these results also highlight the potential applicability of BREGRS in other cancers.

### BREGRS is associated with advanced BLCA stage and bregs’ infiltration levels in the local cohort

**Fig. 6:**
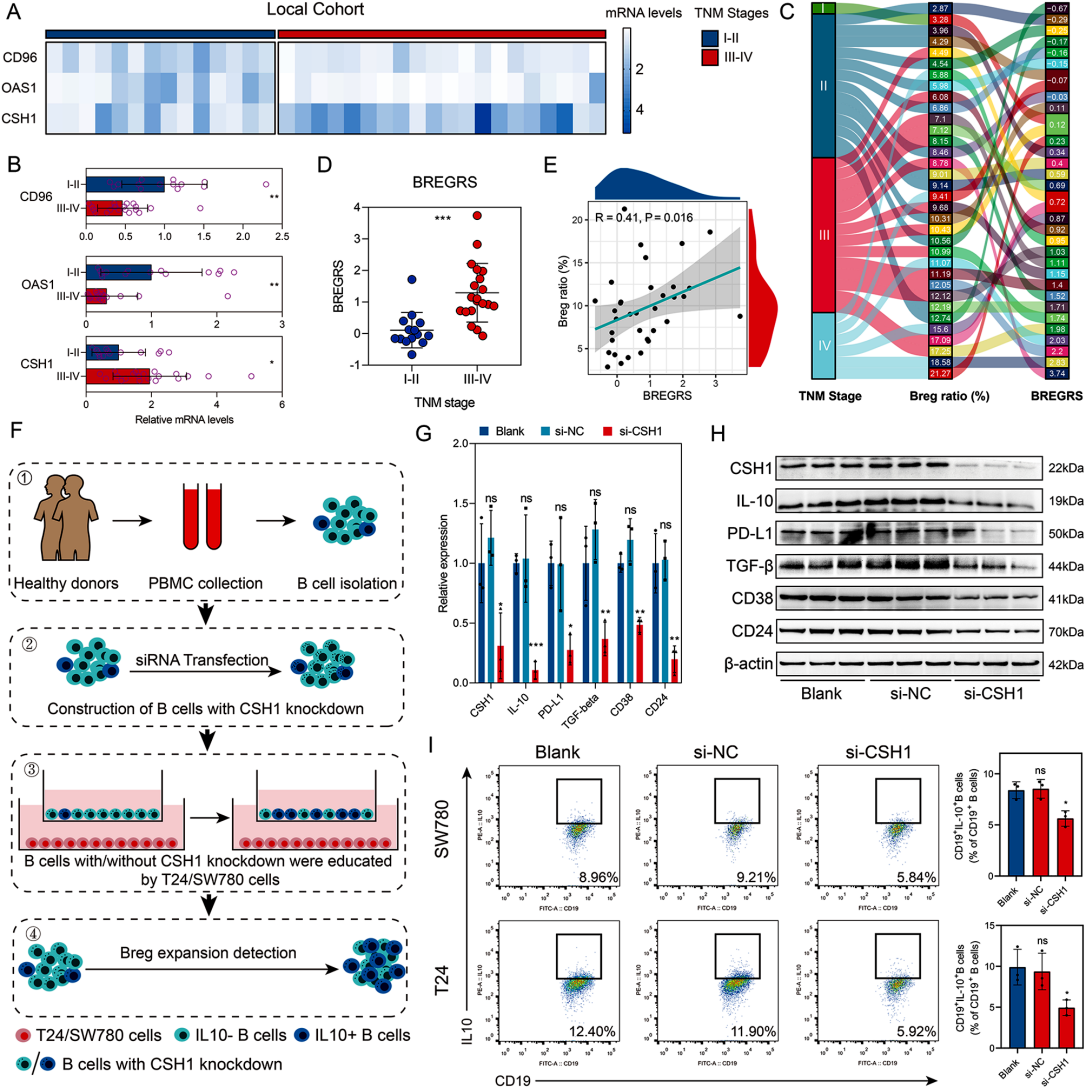
Experimental validation of BREGRS. (**A**) The mRNA levels of CD96, OAS1, and CSH1 were measured in the local cohort. (**B**) The expression difference of CD96, OAS1, and CSH1 was compared between cases with TNM stage I-II and those with TNM stage III-IV. (**C**) The distribution of TNM stages, Bregs’ infiltration proportion, and BREGRS levels were analyzed in the local cohort. (**D**) BREGRS was found to be associated with advanced TNM stages in the local cohort. (**E**) A correlation was observed between BREGRS and the infiltration levels of Bregs. (**F**) An experimental design was implemented to investigate the functions of CSH1 in the expansion of Bregs. (**G**) Knockdown of CSH1 resulted in suppressed mRNA expressions of CSH1, IL10, PD-L1, TGFβ, CD38, and CD24 in B cells. (**H**) Knockdown of CSH1 inhibited the protein expressions of CSH1, IL10, CD24, CD38, PDL1, and TGFβ in B cells. (**I**) A decreased expansion of Bregs was observed in B cells with CSH1 knockdown. *P < 0.05; **P < 0.01; ***P < 0.001

As the calculation of BREGRS relied on mRNA expression levels, we proceeded to perform RT-qPCR experiments to measure the mRNA levels of CD96, OAS1, and CSH1 in 34 BLCA samples obtained from our local hospital (Fig. 6A). The baseline clinicopathological features of the local cohort are displayed in Table S10. Comparing samples with TNM stage I-II to those with TNM stage III-IV, we observed higher expressions of CSH1 (*P* < 0.05) and lower expressions of CD96 and OAS1 (both *P* < 0.01) in the latter group (Fig. 6B), which aligned with the findings from immunohistochemical staining analysis. Subsequently, we calculated the BREGRS for each subject based on the RT-qPCR experiments (Fig. 6C). Notably, a significant increase in BREGRS levels was evident in cases with advanced TNM stages (*P* < 0.001, Fig. 6D), and we observed a positive correlation between BREGRS and Breg infiltration proportion (*R* = 0.41, *P* < 0.05, Fig. 6E).

### Knockdown of CSH1 inhibits the expansion of bregs

After the previous reports on the roles of CD96 and OAS1 in the tumorigenesis of BLCA [30, 31], we decided to focus on further experimental validation of CSH1. We performed a transient transfection assay on B cells isolated from healthy donors’ PBMC using siRNAs targeting CSH1 to accomplish this. Subsequently, these B cells were co-incubated with T24 and SW780 BLCA cells (Fig. 6F). The results of the RT-qPCR experiments indicated that siRNA-2 displayed the most potent inhibitory ability (*P* < 0.01, Fig. S10). Thus it was selected for further analysis. Remarkably, we observed a significant decrease in the expression levels of IL10 (*P* < 0.001), CD24 (*P* < 0.05), CD38 (*P* < 0.01), PDL1 (*P* < 0.05), and TGFβ (*P* < 0.01) in B cells with CSH1-knockdown, both at the mRNA level (Fig. 6G) and protein level (Fig. 6H, Fig. S11). Furthermore, a decrease in the proportion of Breg cells was observed in the B cells with CSH1-knockdown (both *P* < 0.05, Fig. 6I).

### BREGRS is associated with CD8 + T cells’ abundance and functions

**Fig. 7:**
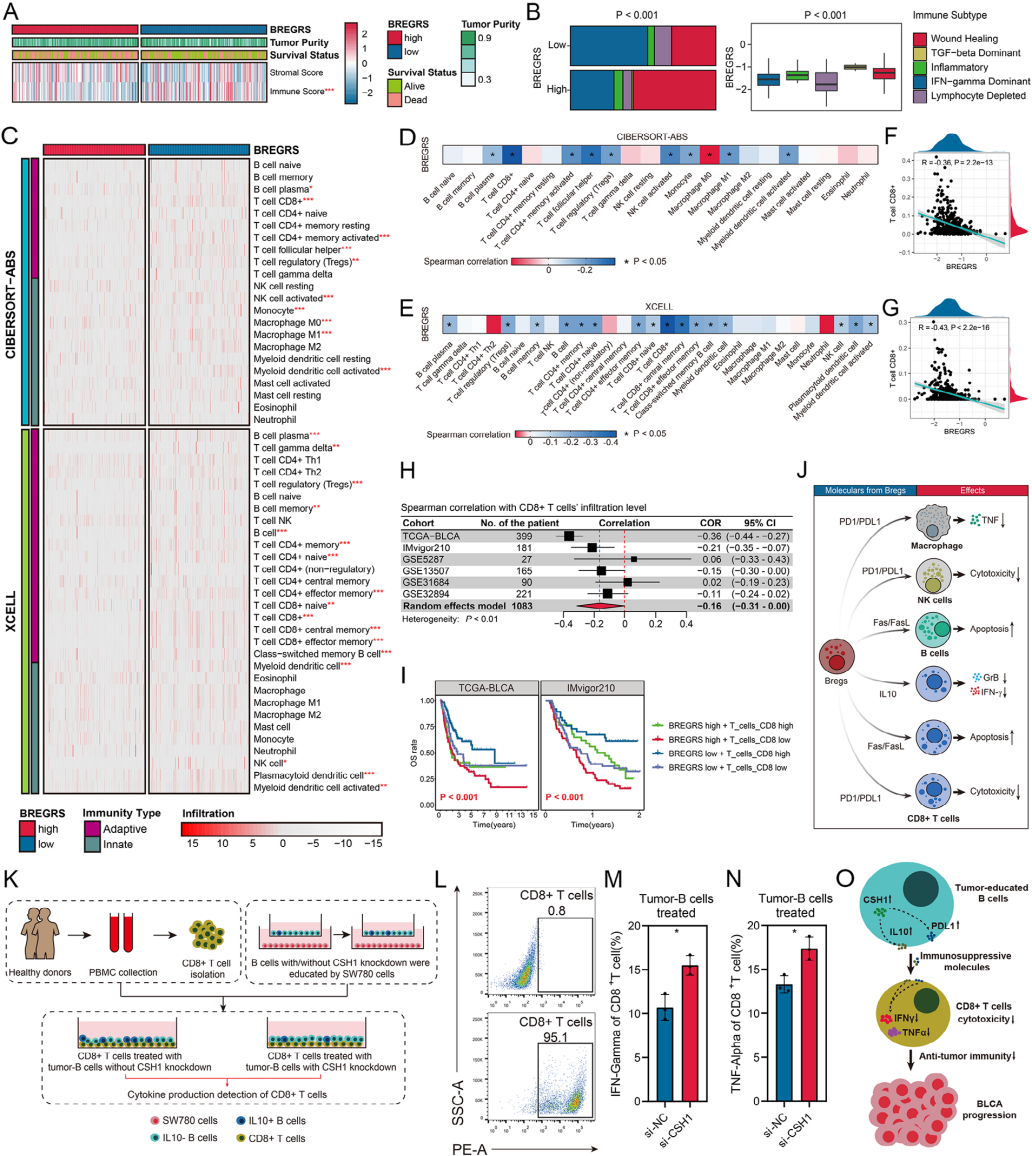
BREGRS was found to be associated with the abundance and cytotoxicity functions of CD8 + T cells. (**A**) BREGRS showed an association with immune component levels, rather than stromal component levels, in the tumor microenvironment of BLCA. (**B**) The association of BREGRS with immune subtypes of BLCA cases. (**C**) Differences in immune cell infiltration were observed between subjects with low and high BREGRS. (**D, E**) The association of BREGRS with immune cell infiltration levels was evaluated using the CIBERSORT-ABS (**D**) and XCELL (**E**) algorithms. (**F, G**) BREGRS exhibited a correlation with the infiltration proportion of CD8 + T cells based on the CIBERSORT-ABS (**F**) and XCELL (**G**) algorithms. (**H**) Meta-analysis was conducted to pool the Spearman correlation coefficient between BREGRS and the infiltration levels of CD8 + T cells. (**I**) Cases with high BREGRS and low infiltration of CD8 + T cells showed worse overall survival. (**J**) An overview of the interactions between Bregs and other immune cells. (**K**) A study design was implemented to investigate the influence of CSH1 expression in B cells on the cytotoxicity functions of CD8 + T cells. (**L**) Purity of CD8 + T cells isolated from PBMC using magnetic beads was assessed through flow cytometry analysis. (**M, N**) Co-incubation of B cells with CSH1 knockdown and SW780 cells education promoted the production of IFNγ (**M**) and TNFα (**N**) by CD8 + T cells. (**O**) CSH1 was found to enhance the expression of immunosuppressive molecules in B cells that were educated by BLCA cells. This ultimately resulted in the expansion of Bregs, a reduction in the cytotoxicity of CD8 + T cells, and the progression of BLCA. *P < 0.05; **P < 0.01; ***P < 0.001

We analyzed the immune landscape to better understand the difference in OS between the high- and low-BREGRS subgroups. In the TCGA-BLCA cohort, cases with high BREGRS exhibited lower immune components (*P* < 0.001, Fig. 7A, Fig. S12A). Similar findings were observed in the IMvigor210 cohort (*P* < 0.01, Fig. S12B). Furthermore, BREGRS was associated with immune subtypes (*P* < 0.001, Fig. 7B) and served as a significant prognostic indicator, particularly in cases with “Lymphocyte Depleted” (HR = 3.51, 95% CI = 1.08–6.82) and “Wound Healing” (HR = 3.25, 95% CI = 1.75–6.03) subtypes Table S17). These results highlight the significant alteration of the tumor immune microenvironment between high- and low-BREGRS cases.

Next, we investigated the infiltration difference of various immune cells between the low- and high-BREGRS subjects using the XCELL and CIBERSORT-ABS algorithms (Fig. 7C, Fig. S13). BREGRS showed a significant negative association with multiple functional immune cells (Fig. 7D-E). Notably, BREGRS exhibited the strongest association with CD8 + T cell infiltration, as observed in both the CIBERSORT-ABS (*R* = -0.36, *P* < 0.001, Fig. 7F) and XCELL (*R* = -0.43, *P* < 0.001, Fig. 7G) analyses. To validate this finding, we evaluated the immune cell infiltration proportion in the IMvigor210, GSE5287, GSE13507, GSE31684, and GSE32894 cohorts using the CIBERSORT-ABS algorithm (Fig. S14). A meta-analysis was conducted to pool the Spearman correlation coefficients between BREGRS and CD8 + T cell infiltration levels in these cohorts (pooled *R* = -0.16, 95% CI = -0.31-0.00, *P* < 0.05, Fig. 7H). At a pan-cancer level, BREGRS was negatively associated with CD8 + T cell infiltration proportion in 19 cancers, with no positive correlation observed (all *P* < 0.05, Fig. S15). Additionally, further analysis indicated that cases with high BREGRS and low CD8 + T cell infiltration had the poorest survival (both *P* < 0.001, Fig. 7I). However, it is worth mentioning that BREGRS served as a significant prognostic indicator in both low- (pooled HR = 2.03, 95% CI = 1.54–2.69) and high-CD8 + T cell infiltration levels (pooled HR = 2.12, 95% CI = 1.54–2.90) (Fig. S16), suggesting that the limited abundance of CD8 + T cells cannot fully account for the unfavorable prognoses of the high-BREGRS subjects.

Bregs were previously reported to suppress the cytotoxicity of CD8 + T cells via the secretion of IL10 and PDL1 (Fig. 7J). Therefore, we then investigate the association of BREGRS with the normal functions of CD8 + T cells. We isolated CD8 + T cells from healthy individuals’ PBMCs using magnetic beads and co-incubated them with educated B cells with or without CSH1 knockdown (Fig. 7K). The efficacy of CD8 + T cell isolation was verified by flow cytometry (Fig. 7L). Importantly, increased production of IFNγ (*P* < 0.05, Fig. 7M) and TNFα (*P* < 0.05, Fig. 7N) was observed in CD8 + T cells co-incubated with educated B cells with CSH1 knockdown. Overall, CSH1 promoted the expressions of immunosuppressive molecules represented by IL10 and PD-L1 and thus inhibited the cytotoxicity of effector T cells, leading to the progression of BLCA (Fig. 7O).

### BREGRS is a promising tool to evaluate the immunotherapeutic sensitivity

**Fig. 8:**
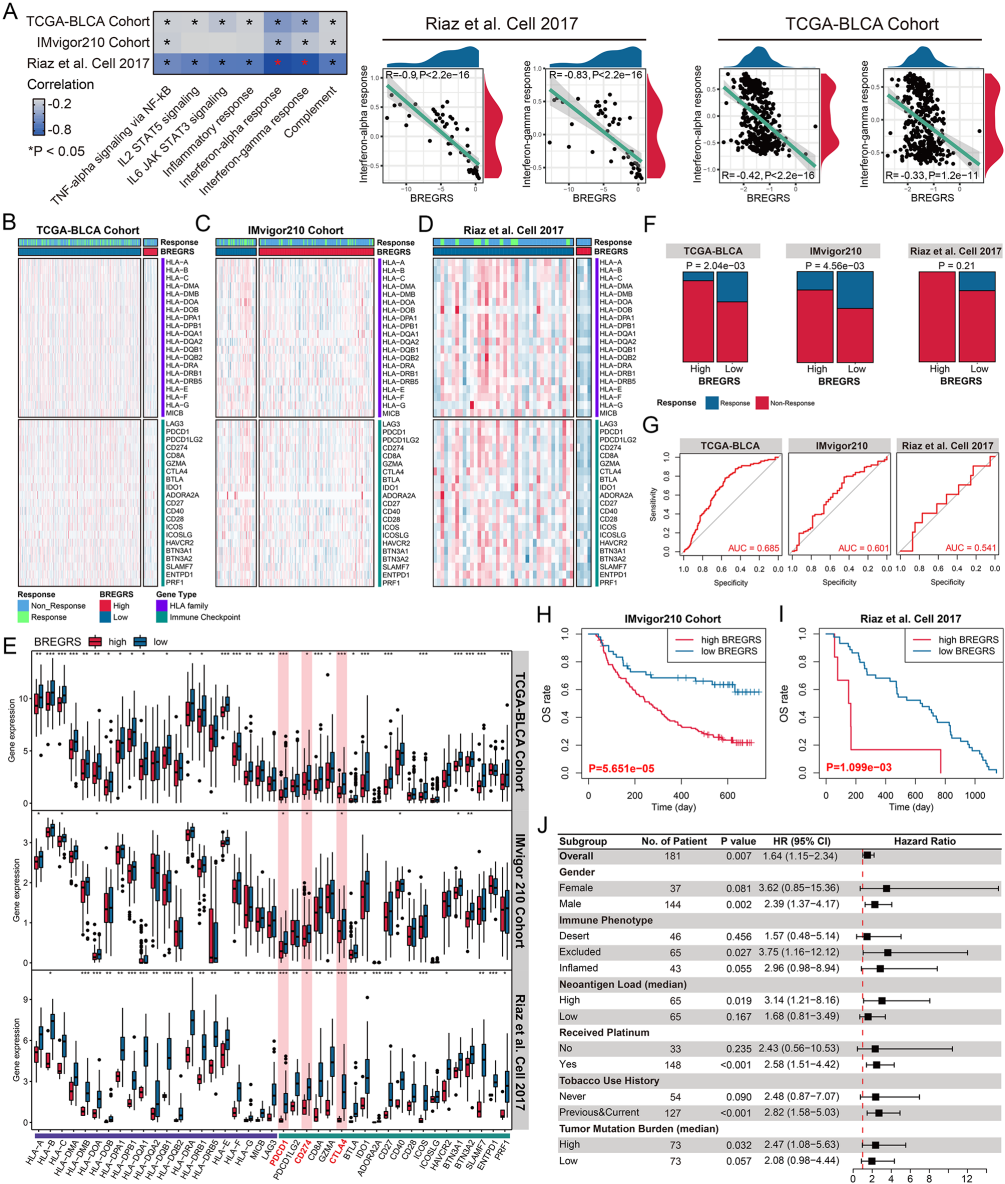
BREGRS is a promising tool to evaluate the immunotherapeutic response. (**A**) The association of BREGRS with the response levels of immune-related pathways. (**B, D**) The expression level of the HLA family and immune checkpoint genes in the TCGA-BLCA (**B**), IMvigor210 (**C**), and Riaz (**D**) cohorts. (**E**) The expression difference of the HLA family and immune checkpoint genes between the low- and high-BREGRS subjects. (**F**) The association between the BREGRS stratification and immunotherapeutic response. (**G**) ROC analyses indicating the predictive ability of BREGRS for the response to immunotherapy. (**H, I**) Cases with high BREGRS suffering unfavorable OS both in the IMvigor210 (**H**) and Riaz (**I**) cohorts. (**J**) Subgroup analyses of BREGRS in the IMvigor210 cohort. HLA, human leukocyte antigen; *P < 0.05; **P < 0.01; ***P < 0.001

The enrichment of immune-related pathways demonstrates a strong negative association between BREGRS and the IFN response (all *P* < 0.001, Fig. 8A). Previous research has shown that elevated levels of IFNγ are associated with favourable clinical outcomes in cancer patients undergoing anti-PD1/PDL1 treatment and are closely linked to PDL1 expression [32, 33]. This inspired us to investigate the predictive potential of BREGRS in immunotherapeutic response.

Since the Riaz cohort consisted of individuals with advanced melanoma, we did not use the same cut-off value mentioned earlier to divide the low- and high-BREGRS subgroups. Instead, we employed X-tile software. We found that BREGRS is negatively associated with multiple HLA family and immune checkpoint genes, particularly those already targeted in immunotherapy, such as PD1, PDL1, and CTLA4 (all *P* < 0.05, Fig. 8B-E). This suggests that individuals with high BREGRS are less likely to benefit from immunotherapy. We subsequently validated this hypothesis in the TCGA-BLCA cohort (*P* < 0.01), IMvigor210 cohort (*P* < 0.01), and Riaz cohort (*P* > 0.05) (Fig. 8F). The TCGA-BLCA cases were evaluated for immunotherapeutic response using the TIDE algorithm. Although no significant results were observed in the Riaz Cohort, this may be attributed to the limited sample size (*n* = 50) and heterogeneity across different cancers. Nonetheless, all individuals with high BREGRS in the Riaz cohort exhibited resistance to nivolumab treatment (Fig. 8F). ROC analyses were conducted to clarify the predictive ability further (Fig. 8G). Importantly, individuals with high BREGRS had worse OS in both the IMvigor210 (*P* < 0.001, Fig. 8H) and the Riaz cohort (*P* < 0.01, Fig. 8I).

Compared to the established prognosis signatures associated with B cell profiles in BLCA (Table S11), BREGRS demonstrated impressive predictive performance (AUC = 0.685, 95% CI = 0.630 − 0.737), ranking second only to the signatures constructed by Adam et al. (AUC = 0.748, 95% CI = 0.687 − 0.804), in evaluating the immunotherapeutic response in the TCGA-BLCA cohort (Fig. S17). In the IMvigor210 cohort, when compared to the reported biomarkers for immunotherapy, such as PD1, PDL1, and tumor mutation burden (TMB) [34], BREGRS exhibited a high predictive ability (HR = 2.52, 95% CI = 1.44–4.38), ranking second to PD1 (HR = 3.71, 95% CI = 1.80–7.69) and surpassing TMB (HR = 2.04, 95% CI = 1.36–3.07) and PDL1 (HR = 2.21, 95% CI = 1.46–3.45), in terms of OS (Fig. S18). This was determined through the univariate Cox regression analysis after excluding subjects with missing variables, and the optimal cut-off values were identified using the X-tile software.

Subgroup analysis in the IMvigor210 cohort revealed that BREGRS was a significant prognostic predictor, particularly in males (HR = 2.39, 95% CI = 1.37–4.17), those with excluded immune subtype (HR = 3.75, 95% CI = 1.16–12.12), those with high neoantigen load (HR = 3.14, 95% CI = 1.21–8.16), those undergoing platinum treatment (HR = 2.58, 95% CI = 1.51–4.42), previous and current smokers (HR = 2.82, 95% CI = 1.58–5.03), and those with high TMB (HR = 2.47, 95% CI = 1.08–5.63) (all *P* < 0.05, Fig. 8J). In conclusion, BREGRS shows promise as a tool for evaluating immunotherapeutic sensitivity in BLCA.

### Mutational landscape associated with BREGRS

**Fig. 9:**
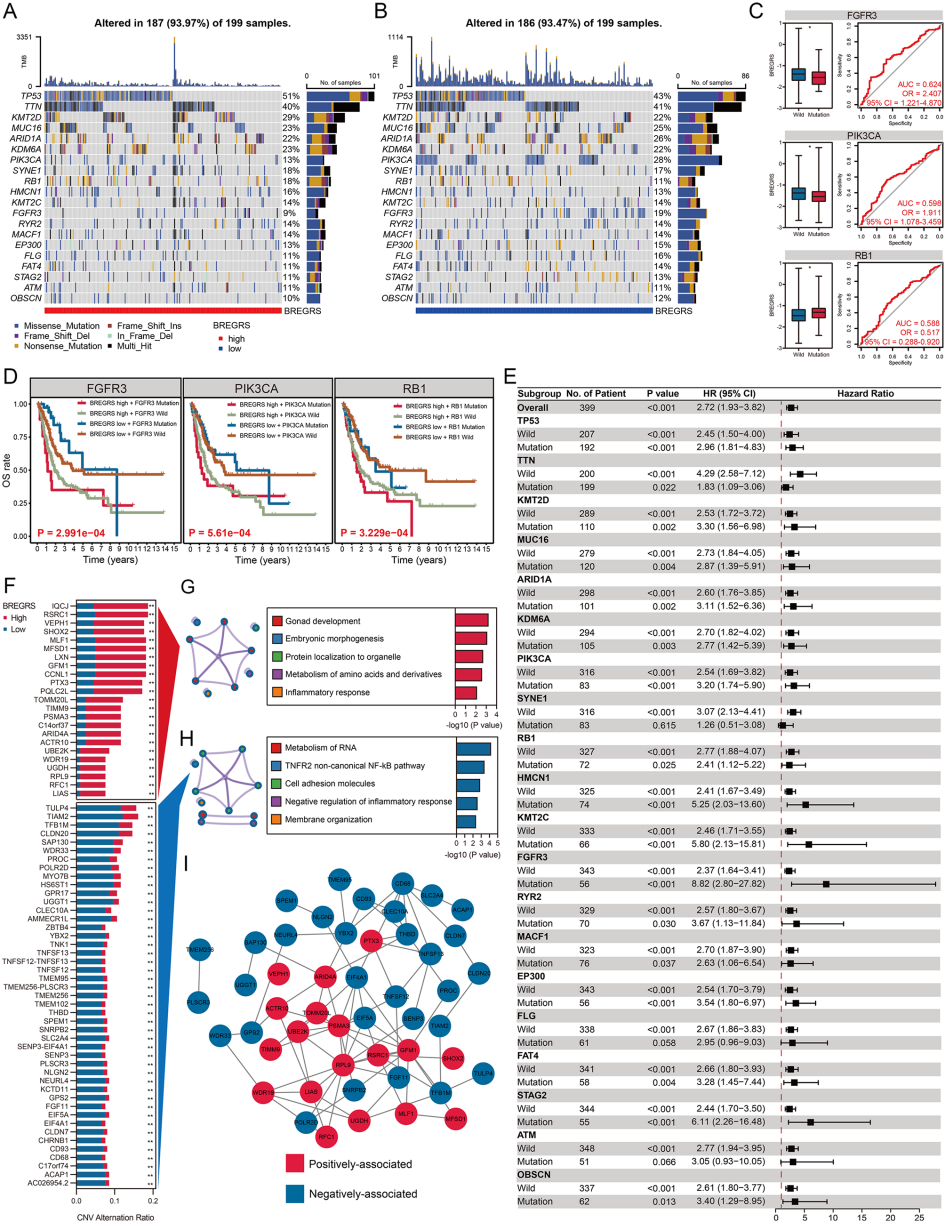
The mutational landscape associated with BREGRS. (**A, B**) The mutational frequency and subtypes of the Top 20 genes with the highest mutational frequency in low- (**A**) and high- (**B**) BREGRS subjects from the TCGA-BLCA cohort. (**C**) BREGRS significantly predicts the mutation statuses of FGFR3, PIK3CA, and RB1. (**D**) Cases with high BREGRS and mutations in FGFR3, PIK3CA, or RB1 exhibit the worst OS. (**E**) Subgroup analysis demonstrates the prognostic value of BREGRS in BLCA cases with different gene mutation statuses. (**F**) The association between BREGRS stratification and copy number variations (CNVs) of specific genes. (**G, H**) Functional annotation of positively-associated (**G**) and negatively-associated (**H**) genes based on CNV frequency. (**I**) A PPI network constructed to reveal the potential interactions among the BREGRS-associated genes based on CNV alterations. CNV, Copy Number Variation; *P < 0.05; **P < 0.01; ***P < 0.001

In order to gain a deeper understanding of the disparities in biological mechanisms between low- and high-BREGRS cases, we examined gene mutation patterns across these subjects. We focused on the top 20 genes with the highest mutation rates in the TCGA-BLCA cohort and investigated the frequency and types of mutations of these genes in the high- (Fig. 9A) and low-BREGRS (Fig. 9B) subgroups. Through the utilization of univariate logistic regression analysis, we were able to identify significant associations between BREGRS and mutations in FGFR3 (Odds Ratio [OR] = 2.41, 95% CI = 1.22–4.87), PIK3CA (OR = 1.91, 95% CI = 1.08–3.46), and RB1 (OR = 0.52, 95% CI = 0.29–0.92) (all *P* < 0.05, Table S18, Fig. 9C). Furthermore, it was observed that high-BREGRS subjects with mutations in these genes exhibited the poorest overall survival (all *P* < 0.001, Fig. 9D). Additional analysis demonstrated that BREGRS served as a significant prognostic biomarker in almost all cases, except for subjects with SYNE1^mut^, FLG ^mut^, and ATM ^mut^ (*P* > 0.05, Fig. 9E). Notably, significant alterations in the Copy Number Variation (CNV) of specific genes were observed between low- and high-BREGRS subjects (Fig. 9F, Table S19). The functional annotation of these genes revealed their involvement in the inflammatory response, both in terms of positively associated (Fig. 9G, Table S20) and negatively associated (Fig. 9H, Table S21) genes based on CNV frequency. Furthermore, the PPI network shed light on tight interactions among these genes (Fig. 9I).

## Discussion

Effector T cells, known for their ability to eliminate tumor cells directly, have been a major focus of tumor immunity research. However, T cell-based effector mechanisms alone are insufficient to combat the adaptive capabilities of malignant cancers [34]. Researchers are now emphasising more on understanding the mechanisms by which cancer cells evade the influence of effector T cells, leading to the discovery of a subset of B cells known as Bregs. Bregs have potent immunosuppressive capabilities and effectively suppress immune surveillance in cancers [35]. Despite their documented roles in multiple cancers, the specific functions and prognostic value of Bregs in BLCA remain insufficiently studied. In this study, we aimed to elucidate the pro-tumor nature of Bregs in BLCA by evaluating their levels in clinical samples and conducting in vitro experiments. Our findings revealed a significant correlation between the infiltration proportion of Bregs and advanced tumor stages, highlighting the importance of understanding Bregs in BLCA. Additionally, we established a Breg-related gene signature that served as a prognostic and immunotherapeutic efficacy indicator. The gene signature was rigorously validated and showed a significant association with prognosis and Breg infiltration levels in BLCA. Furthermore, our study identified three genes, CD96, OAS1, and CSH1, that showed associations with both prognosis and Breg infiltration levels in BLCA. We focused on further experimental validation of CSH1 and found that it promotes the expansion of Bregs during co-incubation with BLCA cells. Our findings also revealed a robust association between the gene signature and CD8 + T cells, suggesting its potential as an indicator of effector T cell abundance and function. Moreover, the gene signature emerged as a significant predictor for immunotherapeutic sensitivity in cohorts undergoing anti-PD1/PDL1 treatment.

The identification of novel biomarkers has been a prominent focus in oncology, particularly with the significant advancements in genomic sequencing and big-data analysis methods [36, 37]. Numerous attempts have been made to predict the prognosis of BLCA, particularly based on the genomic features of functional immune cells such as T cells [38], NK cells [39], and macrophages [40], leading to our better understanding of the mechanisms and offering useful tools in clinical practice. However, it is important to acknowledge that no immune cell-associated multi-gene signature has been recommended in clinical practice guidelines [41], suggesting that current efforts are insufficient. In this study, we have successfully established a prognostic and immunotherapeutic efficacy indicator in the form of a Breg-related gene signature, which was rigorously validated through meta-analysis and in vitro experiments. Importantly, we compared the prognosis predictive ability of the Breg-related gene signature with the established risk signature associated with B cells’ profiles [26–29], and the results suggested that BREGRS was of the strongest predictive ability for OS in the TCGA-BLCA cohort. Besides, BREGRS emerged as a significant predictor for immunotherapeutic sensitivity within immunotherapeutic cohorts. BREGRS exhibited superior performance compared to widely-accepted biomarkers such as PDL1 and TMB. In summary, this study introduces a novel biomarker, BREGRS, which holds considerable potential for predicting the prognosis and immunotherapeutic response in BLCA. Our study helped to guide the personalized treatment and thus improve the prognosis of BLCA subjects.

In this study, we identified three genes, namely CD96, OAS1, and CSH1, which showed associations with both prognosis and infiltration levels of Bregs in BLCA (all *P* < 0.05). Since the roles of CD96 and OAS1 in BLCA have been previously reported [30, 31], we focused on further experimental validation of CSH1. Our findings demonstrated a significant inhibition of Bregs’ expansion upon knockdown of CSH1 during co-incubation with BLCA cells, suggesting that CSH1 acted as an effective regulator for Bregs’ expansion ability. CSH1, also known as Chorionic Somatomammotropin Hormone 1, is primarily synthesized by the placenta during pregnancy [42]. Interestingly, immunohistochemical staining revealed that protein levels of CSH1 were undetectable in nearly half of the BLCA samples in our local cohort, similar to previous observations in breast cancer [43]. However, in contrast to its limited presence in tumor cells, CSH1 levels were significantly elevated in Bregs, which was found to promote their expansion. These findings suggest that CSH1 may exert its pro-tumor functions through regulating in Bregs’ differentiation, rather than exerting direct effects on tumor cells. In summary, our findings highlight the potential significance of CSH1 in the regulation of Bregs and its implications in BLCA progression for the first time and targeting CSH1 in Bregs seems a promising treatment strategy for BLCA cases.

Nevertheless, it is important to acknowledge certain limitations of this study. Firstly, our investigation focused solely on the impact of Bregs on BLCA cells. As previously mentioned, Bregs have the potential to interact with various cell types within the tumor microenvironment, thereby exerting pro-tumor effects. Future studies should delve into these interactions to gain a more comprehensive understanding. Secondly, despite the validation of BREGRS efficacy across multiple cohorts, in vitro experiments, and local clinical samples, its clinical application is hindered by the retrospective nature of this study. Prospective studies are necessary to further evaluate its utility in a real-time clinical setting. Thirdly, although the regulatory role of CSH1 in Bregs expansion has been confirmed, the underlying biological mechanisms remain unclear. Further investigations are warranted to elucidate these intricate processes.

## Conclusion

The infiltration of Bregs showed a strong correlation with the advanced BLCA stage, and Bregs played a direct role in promoting the migration and invasion of BLCA cells, underscoring their contribution to BLCA progression. In addition, we successfully developed a robust multiple-gene signature associated with Bregs, which holds great promise as a prognostic indicator and a valuable tool for predicting immunotherapeutic response in BLCA. This signature provides valuable insights into the underlying biological mechanisms and serves as a practical clinical tool for guiding personalized treatment strategies.

### Electronic supplementary material

Below is the link to the electronic supplementary material.

Supplementary Material 1

Supplementary Material 2

Supplementary Material 3

## Data Availability

All remaining data and materials are available from the authors upon reasonable request.

## Abbreviations

Breg: regulatory B cell
BLCA: bladder cancer
NMIBC: non-muscle-invasive bladder cancer
MIBC: muscle-invasive bladder cancer
Treg: regulatory T cell
PBMC: peripheral blood mononuclear cell
TCGA: The Cancer Genome Atlas
GEO: Gene Expression Omnibus
OS: Overall Survival
LASSO: Least Absolute Shrinkage and Selection Operator
TNM: tumor-node-metastasis
GO: Gene Ontology
DEG: differentially-expressed gene
GSEA: Gene Set Enrichment Analysis
BREGRS: Breg-related score
HR: Hazard Ratio
RT-qPCR: real-time quantitative PCR
siRNA: mall-interference RNA
MSigDB: Molecular Signatures Database
TIDE: Tumor Immune Dysfunction and Exclusion
PPI: protein-protein interaction
ssGSEA: single-sample Gene Set Enrichment Analysis
FDR: False Discovery Rate
NES: Normalized Enrichment Score
ROC: Receiver Operating Characteristic
AUC: Area Under Curve
CI: Confidence Interval
HL: human leukocyte antigen
TMB: tumor mutation burden
OR: Odds Ratio
CNV: Copy Number Variation
