# Supplementary material for: Investigating the impact of regulatory B cells and regulatory B cell-related genes on bladder cancer progression and immunotherapeutic sensitivity
Source: J Exp Clin Cancer Res. 2024 Apr 2;43:101. doi: 10.1186/s13046-024-03017-8 (PMC10985985; doi:10.1186/s13046-024-03017-8)
Supplement: Supplementary file 2 — Supplementary Material 2 [file 13046_2024_3017_MOESM2_ESM.docx]

**Supplementary File 2: Materials and Methods**

**Content**

[1. Experimental study 1](#_Toc159446671)

[1.1 BLCA sample collection 1](#_Toc159446672)

[1.2 Immunohistochemical staining 2](#_Toc159446673)

[1.3 Tumor cell culture 3](#_Toc159446674)

[1.4 Cells isolation from peripheral blood 3](#_Toc159446675)

[1.5 Co-incubation assay to achieve educated B cells 3](#_Toc159446676)

[1.6 Flow cytometry analysis 4](#_Toc159446677)

[1.7 Wound healing assay 4](#_Toc159446678)

[1.8 Transwell assay 5](#_Toc159446679)

[1.9 Transfection of siRNAs in B cells 5](#_Toc159446680)

[1.10 Real-time quantitative PCR (RT-qPCR) 6](#_Toc159446681)

[1.11 Western blot 6](#_Toc159446682)

[2. Bioinformatical analyses 7](#_Toc159446683)

[2.1 Identifying the genes associated with BLCA 7](#_Toc159446684)

[2.2 Cohorts and data processing 7](#_Toc159446685)

[2.3 Collection of regulatory B cell (Breg)-related genes 9](#_Toc159446686)

[2.4 Functional enrichment and Protein-Protein Interaction (PPI) network construction 10](#_Toc159446687)

[2.5 Gene Set Enrichment Analysis (GSEA), Gene Set Variation Analysis (GSVA), and single-sample GSEA (ssGSEA) 11](#_Toc159446688)

[2.6 Breg-related gene signature construction 11](#_Toc159446689)

[2.7 Immunogenetic analysis 12](#_Toc159446690)

[2.8 Survival difference detection and meta-analyses 12](#_Toc159446691)

[2.9 Pan-cancer analyses 13](#_Toc159446692)

[3. Statistical analyses 13](#_Toc159446693)

[4. References 14](#_Toc159446694)

# 1. Experimental study

## 1.1 BLCA sample collection

Between May 2021 and November 2022, tumor tissues and corresponding paracancerous tissues were collected from 34 subjects who underwent cystectomy and were pathologically diagnosed with bladder cancer (BLCA). The collection was conducted at the Nanfang Hospital of Southern Medical University. Tumor tissues were obtained from the tumor center, while paracancerous tissues were extracted from areas >3 cm away from the tumor margin. None of the patients had received preoperative chemotherapy or radiotherapy. Clinicopathological features, such as age, gender, pathological T stages, pathological N stages, M stages, and TNM stages, were recorded from the Electronic Medical Record System at Nanfang Hospital. The study protocol was reviewed and approved by the Ethics Committee of Nanfang Hospital of Southern Medical University.

## 1.2 Immunohistochemical staining

The dual immunohistochemistry protocol utilized in this study was previously described (1). In summary, tumor specimens were baked at 65°C for 2 hours. The slides were then subjected to heat in EDTA repair solution at pH 9.0 in boiling water for 20 minutes, followed by incubation with 10% goat serum at 37°C for 45 minutes. Subsequently, the slides were incubated overnight at 4°C with anti-CD19 antibodies (Origene, USA) and anti-IL10 antibodies (ABclonal, China). HRP-labeled and AP-labeled secondary antibodies were applied and incubated at 37°C for 45 minutes. Positive staining was detected using DAB and Vector blue staining solution.

For staining of CSH1, CD96, and OAS1 in BLCA tissue, a similar protocol as described above was employed. After antigenic closure with 10% goat serum, the slides were incubated overnight at 4°C with anti-CD96 antibodies (Abcam, USA), anti-OAS1 antibodies (SAB Signalway Antibody, China), or anti-CSH1 antibodies (ABclonal, China). HRP-labeled secondary antibodies were applied and incubated at 37°C for 45 minutes. Finally, positive staining was detected using DAB staining solution. The analyses on the intensity of the immunohistochemical staining were based on Image-Pro Plus software (version 6.0, USA).

## 1.3 Tumor cell culture

The BLCA cell lines T24 and SW780 were procured from the American Type Culture Collection (ATCC, USA). These cells were cultivated in DMEM medium (Corning, USA) supplemented with 10% fetal bovine serum (Thermo Fisher Scientific, USA), 100 µg/ml penicillin, and 100 µg/ml streptomycin (Beyotime Institute of Biotechnology, China) at a temperature of 37°C in a humidified atmosphere containing 5% CO_2_.

## 1.4 Cells isolation from peripheral blood

Peripheral blood mononuclear cells (PBMCs) were obtained from healthy donor blood samples provided by BRK company (Guangzhou, China). B cells and their corresponding CD8+ T cells were isolated from PBMCs using the CD19+ B Cell Positive Isolation Kit (StemCell Technologies, USA) and CD8+ T Cell Isolation Kit (Miltenyi Biotech, CA), respectively, following the manufacturers' protocol. The isolation efficacy of these magnetic beads was detected using the flow cytometry.

## 1.5 Co-incubation assay to achieve educated B cells

Tumor/B cells co-incubation assays were performed using the transwell system (0.4 um pores, LABSELECT, China). Before constructing the co-culture system, BLCA cells (T24 and SW780) were plated into the lower chamber and cultured to 50% density. CD19+ B cells were isolated as described above, 2 x 10^5^ B cells and medium containing 10% FBS were further placed in the upper chamber with adding IL4 (10ng/ml, PeproTech, USA) and CD40L (100ng/ml, PeproTech, USA). Next, B cells were co-incubated with T24 and SW780 cells for 3 days.

## 1.6 Flow cytometry analysis

The purity of B cells and CD8+ T cells was assessed by staining with anti-CD19-FITC (BioLegend, clone HIB19, CA) and anti-CD8a-PerCP-cy5.5 (BioLegend, clone HIT8a, CA) respectively. Bregs were identified by staining with anti-CD19-FITC (BioLegend, clone HIB19, CA) and anti-IL10-PE (BioLegend, clone JES3-19F1, CA). The cytokines produced by CD8+ T cells were detected using anti-TNFα (BioLegend, clone MAb11, CA) and anti-IFNγ (Elabscience, clone B27, CA). Intracellular cytokine production was measured by re-treating cells with GolgiStop (BD Biosciences, UK) for 4-6 hours, followed by intracellular staining using the Fixation/Permeabilization Solution Kit (BD Biosciences, UK). Finally, data were analyzed using FlowJo software (version 7.6.5, Tree Star, USA).

## 1.7 Wound healing assay

T24 and SW780 cells were seeded in 6-well plates and incubated until they reached 70% confluence. A wound was created by gently scratching the cell monolayer using a pipette tip, and the cells were then cultured for 24 hours. Images were captured at 0 and 24 hours, respectively, and the migration distances were quantified using ImageJ software (version 1.52a, National Institutes of Health, USA).

## 1.8 Transwell assay

BLCA cells’ invasion ability was measured using the transwell system (8 um pores, LABSELECT, China). Tumor-educated B cells were obtained from the tumor/B cells co-cultured system. Briefly, 2 x 10^5^ B cells were seeded in the lower chamber with adding IL4 (10 ng/ml) and CD40L (100 ng/ml). and 1 x 10^5^ BLCA cells and 10% FBS containing media were then plated in the upper chamber. After 24h incubation, the cells on the upper surfaces of the inserts were removed, and cells on the lower surface of the membrane were fixed with 100% methanol and stained with 0.1% crystal violet. Positive cells were counted from at least six randomized fields of view at 200x magnification and the averaged value was obtained.

## 1.9 Transfection of siRNAs in B cells

Three small interfering RNAs (siRNAs) targeting CSH1 and their corresponding interfering control siRNAs (universal negative control) were obtained from IGE Biotechnology (Guangzhou, China). The lyophilized oligonucleotides were reconstituted in a 20 μM stock solution using aqueous DEPC. Subsequently, the CSH1 siRNAs were transfected into the B cells using lipofectamine 3000 (Invitrogen, USA) following the manufacturer's instructions. The cells were then cultured for 72 hours in preparation for the subsequent steps. The sequences of all siRNAs are provided in Table S3.

## 1.10 Real-time quantitative PCR (RT-qPCR)

Total RNA was isolated from cells and BLCA samples using TRizol (Tiangen, China) following the manufacturer's protocol. Subsequently, cDNA synthesis was performed using the PrimeScript® RT reagent kit (Takara Biotechnology, China). The mRNA levels were quantified using the SYBR Master Mix (EZBioscience, USA). GAPDH was selected as the internal reference gene. The relative gene expression was determined using the ΔΔCt method, a comparative threshold value approach. The primers utilized for RT-qPCR analysis can be found in Table S2.

## 1.11 Western blot

The cells were subjected to protein extraction using RIPA buffer. Equal amounts of total protein were loaded onto a 10% polyacrylamide gel, and subsequent protein separation was achieved through SDS-PAGE. The separated proteins were subsequently transferred to a polyvinylidene fluoride membrane. Following this, the membranes were blocked with 3% non-fat dry milk for 1 hour at room temperature, after which they were incubated overnight at 4 °C with primary antibodies. The primary antibodies employed in this study included anti-IL10 (ABclonal, China), anti-CD96 (Abcam, USA), anti-OAS1 (SAB Signalway Antibody, China), anti-CSH1 (ABclonal, China), anti-PD-L1 (ABclonal, China), anti-TGFβ (ABclonal, China), anti-CD38 (Abcam, USA), and anti-CD24 (Abcam, USA). Subsequently, the blots were incubated with corresponding secondary antibodies, and the protein bands were visualized using an enhanced chemiluminescence (ECL) reagent (NCM Biotech, China) in conjunction with an ECL system (Thermo Fisher Scientific, USA). The results were quantified using ImageJ software (version 1.52a, National Institutes of Health, USA).

# 2. Bioinformatical analyses

## 2.1 Identifying the genes associated with BLCA

We collected the genes that strongly associated with BLCA in the GeneCards database (<https://www.genecards.org/>) with the keyword “bladder cancer”. The Top20 genes sharing the highest relevance score were selected for further analysis.

## 2.2 Cohorts and data processing

The transcription sequencing data in the form of Fragments Per Kilobase of exon model per Million mapped fragments (FPKM) and Overall Survival (OS) information from 399 bladder cancer (BLCA) subjects obtained from The Cancer Genome Atlas (TCGA) project were downloaded from the UCSC Xena website (<http://xena.ucsc.edu/>). The TCGA-BLCA cohort was selected as the training set in this study. Additionally, we obtained the Copy Number Variation (CNV) data of the TCGA-BLCA cohort from the same UCSC Xena database. The mutational frequency and subtypes of the TCGA-BLCA cohort were obtained from the official TCGA website (<https://portal.gdc.cancer.gov/>), analyzed by the varscan software, were visualized using the “maftools” package in R software (version 4.2.0). The routine clinicopathological features, such as age, gender, tumor-node-metastasis (TNM) stages, and tumor grade, were obtained through the “TCGAbiolinks” package in R.

For the validation datasets, we searched the Gene Expression Omnibus (GEO, <https://ncbi.nlm.nih.gov/geo/>) database using the keywords "bladder cancer", "urothelial carcinoma", "urothelial cancer", "BCa", or "BC". Two researchers (Zhou J and Zhou R) independently conducted the search, and any conflicts were resolved through discussion with an expert (Tan W). The validation cohort had to meet the following criteria: (1) The patients should be diagnosed with bladder cancer. (2) The transcription sequencing data, along with the corresponding OS statuses and follow-up duration, should be available from the GEO database or the supplementary materials of the original articles. (3) The dataset must include the expressions of the genes in the established prognosis signature.

Ultimately, we identified 5 BLCA datasets for external validation, namely GSE13507 (n = 165) (2), GSE31684 (n = 90) (3, 4), GSE32894 (n = 221) (5), and GSE5287 (n = 27) (6). More detailed information of these GEO datasets can be found in Supplementary Table 1. In addition to these GEO cohorts, the IMvigor210 cohort, which included 181 BLCA subjects and was obtained from the IMvigor210CoreBiologies package in R, were also included. It is worth noting that the BLCA cases from the IMvigor210 cohort had undergone atezolizumab (an anti-PDL1 reagent) treatment, so this cohort was also included in the analysis of immunotherapeutic sensitivity. Additionally, we analyzed another immunotherapy-associated cohort, the Riaz cohort, which consisted of 50 melanoma patients treated with nivolumab (an anti-PD1 reagent) (7).

The quality control and data processing were conducted using the R software. The transcription sequencing data obtained through RNA sequencing (RNA-seq) was normalized into Transcripts Per Kilobase of exon model per Million mapped reads (TPM) form. To reduce batch effects in the BLCA datasets, we used the “sva” package in R. Patients with less than 30 days of follow-up duration and genes with average expression levels below 0.5 were excluded from the study.

## 2.3 Collection of regulatory B cell (Breg)-related genes

To compile Breg-related genes, we conducted a comprehensive data curation process. This involved sourcing immunosuppressive gene sets from Gene Ontology (GO, <https://geneontology.org/>), selecting the top 100 co-expression genes of IL10, TGFβ, and IL35 through GeneMANIA (<http://genemania.org/>), extracting Breg cell marker genes from CellMarker (<http://bio-bigdata.hrbmu.edu.cn/CellMarker/>), and identifying differentially-expressed genes between IL10- and IL10+ B cells from GEO (GSE50895, <https://ncbi.nlm.nih.gov/geo/>).

The immunosuppressive gene sets obtained from GO encompassed 9 distinct categories as previously reported (8), specifically "GOBP NEGATIVE REGULATION OF INTERLEUKIN 1 PRODUCTION," "GOBP NEGATIVE REGULATION OF INTERLEUKIN 6 PRODUCTION," "GOBP POSITIVE REGULATION OF INTERLEUKIN 10 PRODUCTION," "GOBP NEGATIVE REGULATION OF INTERLEUKIN 12 PRODUCTION," "GOBP NEGATIVE REGULATION OF INTERLEUKIN 17 PRODUCTION," "GOBP NEGATIVE REGULATION OF IMMUNE SYSTEM PROCESS," "GOBP NEGATIVE REGULATION OF INTERFERON GAMMA PRODUCTION," "GOBP POSITIVE REGULATION OF CELLULAR RESPONSE TO TRANSFORMING GROWTH FACTOR BETA STIMULUS," and "GOBP NEGATIVE REGULATION OF TUMOR NECROSIS FACTOR SUPERFAMILY CYTOKINE PRODUCTION."

Since the IL-35 protein is encoded by the genes EBI3 (Epstein-Barr virus-induced gene 3) and IL12A (interleukin 12A), we analyzed the co-expression genes of IL35 based on EBI3 and IL12A on the GeneMANIA database (9, 10).

To identify differentially-expressed genes (DEGs) in the GSE50895 dataset, which consisted of transcription sequencing data from 5 IL10- and 5 IL10+ B cell samples (11), we employed the “limma” package in R. DEGs were selected based on criteria of |logFC| > 0.5 and P < 0.05.

## 2.4 Functional enrichment and Protein-Protein Interaction (PPI) network construction

The specific gene cluster underwent functional enrichment analysis using the Metascape (12) database (<https://metascape.org/gp/index.html#/main/step1>) with default parameters. To explore potential correlations among the genes, a protein-protein interaction (PPI) network was constructed based on the STRING (13) database (<https://cn.string-db.org/>) with a confidence level threshold set at 0.3. The resulting PPI network was visualized using Cytoscape software (version 3.8.0).

## 2.5 Gene Set Enrichment Analysis (GSEA), Gene Set Variation Analysis (GSVA), and single-sample GSEA (ssGSEA)

GSEA analysis was conducted using the GSEA software (version 4.3.2), and only terms with both Nominal P < 0.05 and False Discovery Rate (FDR) < 0.05 were considered statistically significant. To calculate the response levels of immune-related pathways, GSVA analysis was performed using the “GSVA” package in R. The gene sets for immune-related pathways were obtained from the HALLMARK gene sets available in the Molecular Signatures Database (MSigDB, <https://www.gsea-msigdb.org/gsea/msigdb/>). Additionally, ssGSEA analysis was conducted using the “GSVA” package, utilizing the “ssGSEA” function.

## 2.6 Breg-related gene signature construction

LASSO regression with 10-fold cross-validation was employed using the "glmnet" package to identify Breg-related genes significantly associated with overall survival (OS). Simultaneously, random forest analysis was conducted using the "var.select" function in the "randomForestSRC" package (14) to identify significant genes associated with OS. The genes identified by both LASSO and random forest analyses were included in a multivariate Cox regression model using the "survival" package in R. Based on this model, a risk score named Breg-related score (BREGRS) was calculated using the formula: $\mathrm{BREGRS}=\sum_{i=1}^{n} {{Coeff}_{i}*Exp(Gene)}_{i}$, where "Coeff" represents the coefficients of the variables in the Cox regression model and "Exp" denotes the mRNA expression levels of the genes.

## 2.7 Immunogenetic analysis

The ESTIMATE algorithm, which stands for the Estimation of STromal and Immune cells in MAlignant Tumor tissues using Expression data, was employed to quantify the abundance of stromal and immune components within the tumor microenvironment. This analysis was conducted using the "estimate" package (15) in R. Additionally, the infiltration proportions of a diverse range of immune cells within the tumor microenvironment were evaluated using the XCELL and CIBERSORT-ABS algorithms. These algorithms were implemented using the "immunedeconv" package in R. The response to immune checkpoint inhibitor therapy in the TCGA-BLCA cases was predicted using The Tumor Immune Dysfunction and Exclusion (TIDE) algorithm (<http://tide.dfci.harvard.edu>).

## 2.8 Survival difference detection and meta-analyses

Differences in OS across various subgroups were assessed using Kaplan-Meier analysis with log-rank test or two-stage hazard rate comparison. This analysis was conducted using the "TSHRC" package (16) in R. Univariate and multivariate Cox regression analyses were performed using the "survival" package in R. Meta-analyses were conducted to combine the Hazard Ratios (HRs) and Spearman correlation coefficients using the "metagen" and "metacor" functions in the "meta" package in R, respectively. The choice between a random effects model or fixed-effect model was determined based on the results of the heterogeneity test. Specifically, if the heterogeneity test yielded a P value < 0.05, the random effects model was employed; otherwise, the fixed-effect model was utilized.

## 2.9 Pan-cancer analyses

To investigate the prognostic significance of BREGRS in various cancers, pan-cancer analyses were performed utilizing data from the TCGA database. Transcription sequencing data and corresponding follow-up information were obtained from the UCSC Xena website. Additionally, pan-cancer analysis at the single-cell level was conducted using the CancerSEA database (<http://biocc.hrbmu.edu.cn/CancerSEA/>) (17).

# 3. Statistical analyses

Statistical analyses for the entire study were conducted using R software (version 4.2.0). The infiltration difference between BLCA and the corresponding adjacent normal tissues was compared using the paired Student's t-test. Other experimental data were analyzed using Welch's corrected t-test. Additionally, Wilcoxon signed-rank or Kruskal-Wallis tests were employed to elucidate differences in two subgroups and subgroups with a number ≥ 3, respectively. The Spearman correlation analysis was performed using the "cor.test" function in R. Receiver Operating Characteristic (ROC) analyses were conducted using the "pROC" or "timeROC" package. Dimension-reduction algorithms, such as Principal Component Analysis (PCA) and t-distributed Stochastic Neighbor Embedding (t-SNE), were accomplished using the "prcomp" function and the "Rtsne" package in R. Categorical variables were compared using Pearson's chi-square test or Fisher's exact test. Unless otherwise specified, a level of P < 0.05 was considered statistically significant.

# 4. References

1. Hu B, Wang Z, Zeng H, Qi Y, Chen Y, Wang T, et al. Blockade of DC-SIGN(+) Tumor-Associated Macrophages Reactivates Antitumor Immunity and Improves Immunotherapy in Muscle-Invasive Bladder Cancer. Cancer Res. 2020;80(8):1707-19.

2. Lee JS, Leem SH, Lee SY, Kim SC, Park ES, Kim SB, et al. Expression signature of E2F1 and its associated genes predict superficial to invasive progression of bladder tumors. J Clin Oncol. 2010;28(16):2660-7.

3. Riester M, Taylor JM, Feifer A, Koppie T, Rosenberg JE, Downey RJ, et al. Combination of a novel gene expression signature with a clinical nomogram improves the prediction of survival in high-risk bladder cancer. Clin Cancer Res. 2012;18(5):1323-33.

4. Riester M, Werner L, Bellmunt J, Selvarajah S, Guancial EA, Weir BA, et al. Integrative analysis of 1q23.3 copy-number gain in metastatic urothelial carcinoma. Clin Cancer Res. 2014;20(7):1873-83.

5. Sjodahl G, Lauss M, Lovgren K, Chebil G, Gudjonsson S, Veerla S, et al. A molecular taxonomy for urothelial carcinoma. Clin Cancer Res. 2012;18(12):3377-86.

6. Als AB, Dyrskjot L, von der Maase H, Koed K, Mansilla F, Toldbod HE, et al. Emmprin and survivin predict response and survival following cisplatin-containing chemotherapy in patients with advanced bladder cancer. Clin Cancer Res. 2007;13(15 Pt 1):4407-14.

7. Riaz N, Havel JJ, Makarov V, Desrichard A, Urba WJ, Sims JS, et al. Tumor and Microenvironment Evolution during Immunotherapy with Nivolumab. Cell. 2017;171(4):934-49 e16.

8. Yang SY, Long J, Huang MX, Luo PY, Bian ZH, Xu YF, et al. Characterization of Organ-Specific Regulatory B Cells Using Single-Cell RNA Sequencing. Front Immunol. 2021;12:711980.

9. Zhang X, Zhang Z, Ju M, Li J, Jing Y, Zhao Y, et al. Pretreatment with interleukin 35-engineered mesenchymal stem cells protected against lipopolysaccharide-induced acute lung injury via pulmonary inflammation suppression. Inflammopharmacology. 2020;28(5):1269-81.

10. Franz M, Rodriguez H, Lopes C, Zuberi K, Montojo J, Bader GD, et al. GeneMANIA update 2018. Nucleic Acids Res. 2018;46(W1):W60-W4.

11. Lin W, Cerny D, Chua E, Duan K, Yi JT, Shadan NB, et al. Human regulatory B cells combine phenotypic and genetic hallmarks with a distinct differentiation fate. J Immunol. 2014;193(5):2258-66.

12. Zhou Y, Zhou B, Pache L, Chang M, Khodabakhshi AH, Tanaseichuk O, et al. Metascape provides a biologist-oriented resource for the analysis of systems-level datasets. Nat Commun. 2019;10(1):1523.

13. Szklarczyk D, Kirsch R, Koutrouli M, Nastou K, Mehryary F, Hachilif R, et al. The STRING database in 2023: protein-protein association networks and functional enrichment analyses for any sequenced genome of interest. Nucleic Acids Res. 2023;51(D1):D638-D46.

14. Qi X, Ge Y, Yang A, Liu Y, Wang Q, Wu G. Potential value of mitochondrial regulatory pathways in the clinical application of clear cell renal cell carcinoma: a machine learning-based study. J Cancer Res Clin Oncol. 2023;149(19):17015-26.

15. Yoshihara K, Shahmoradgoli M, Martinez E, Vegesna R, Kim H, Torres-Garcia W, et al. Inferring tumour purity and stromal and immune cell admixture from expression data. Nat Commun. 2013;4:2612.

16. Dormuth I, Liu T, Xu J, Pauly M, Ditzhaus M. A comparative study to alternatives to the log-rank test. Contemp Clin Trials. 2023;128:107165.

17. Yuan H, Yan M, Zhang G, Liu W, Deng C, Liao G, et al. CancerSEA: a cancer single-cell state atlas. Nucleic Acids Res. 2019;47(D1):D900-D8.
