# Supplementary material for: Investigating the impact of regulatory B cells and regulatory B cell-related genes on bladder cancer progression and immunotherapeutic sensitivity
Source: J Exp Clin Cancer Res. 2024 Apr 2;43:101. doi: 10.1186/s13046-024-03017-8 (PMC10985985; doi:10.1186/s13046-024-03017-8)
Supplement: Supplementary file 3 — Supplementary Material 3 [file 13046_2024_3017_MOESM3_ESM.docx]

**Supplementary Figures**

**Content**

[Fig. S1 1](#_Toc159439734)

[Fig. S2 2](#_Toc159439735)

[Fig. S3 3](#_Toc159439736)

[Fig. S4 4](#_Toc159439737)

[Fig. S5 5](#_Toc159439738)

[Fig. S6 5](#_Toc159439739)

[Fig. S7 5](#_Toc159439740)

[Fig. S8 6](#_Toc159439741)

[Fig. S9 6](#_Toc159439742)

[Fig. S10 7](#_Toc159439743)

[Fig. S11 7](#_Toc159439744)

[Fig. S12 7](#_Toc159439745)

[Fig. S13 8](#_Toc159439746)

[Fig. S14 9](#_Toc159439747)

[Fig. S15 10](#_Toc159439748)

[Fig. S16 11](#_Toc159439749)

[Fig. S17 11](#_Toc159439750)

[Fig. S18 12](#_Toc159439751)


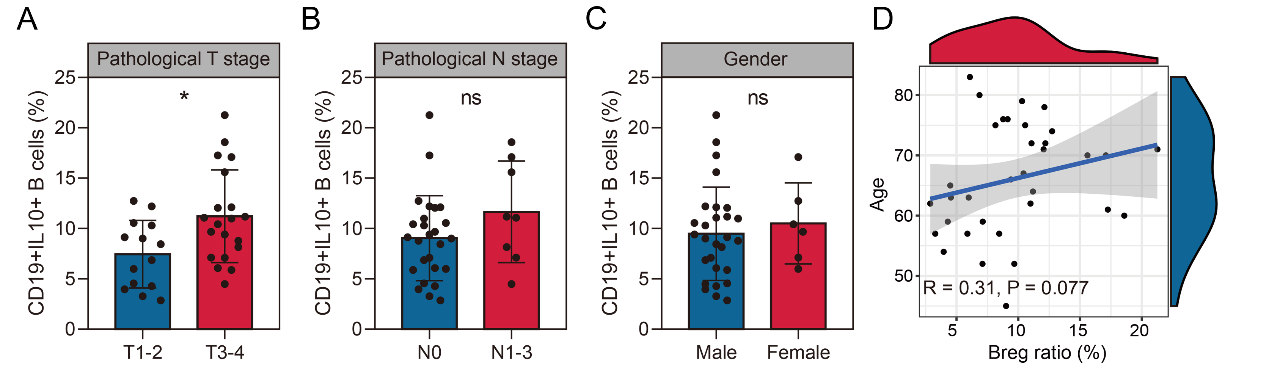


Fig. S1 Association of Bregs' infiltration levels with pathological T stages (**A**), pathological N stages (**B**), gender (**C**), and age (**D**) in the local cohort. *Breg, regulatory B cells; *P < 0.05; ns, not significant.*


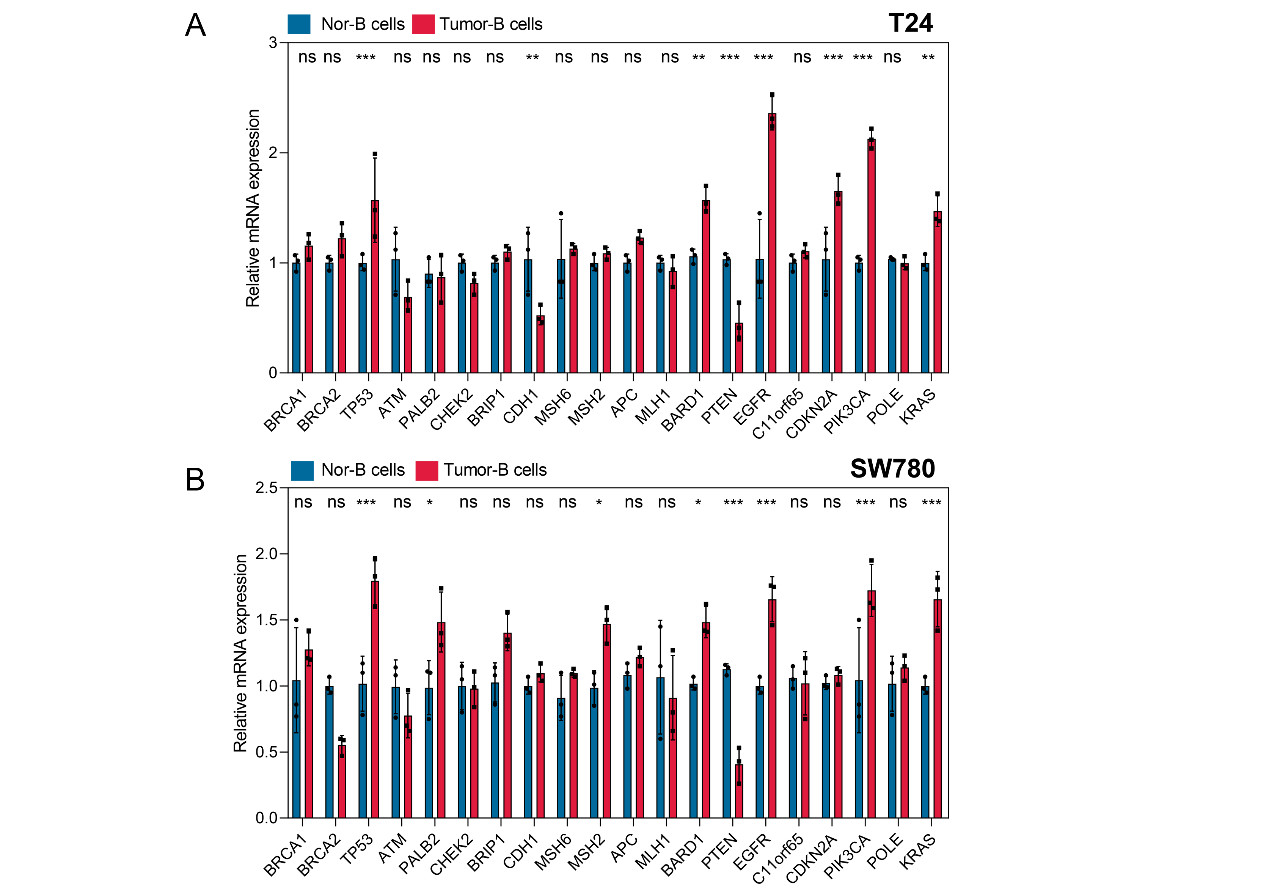


Fig. S2 Difference expressions of Top 20 genes most associated with BLCA in T24 (**A**) and SW780 (**B**) cells treated with or without tumor-induced B cells. **P < 0.05; **P < 0.01; ***P < 0.001; ns, not significance.*


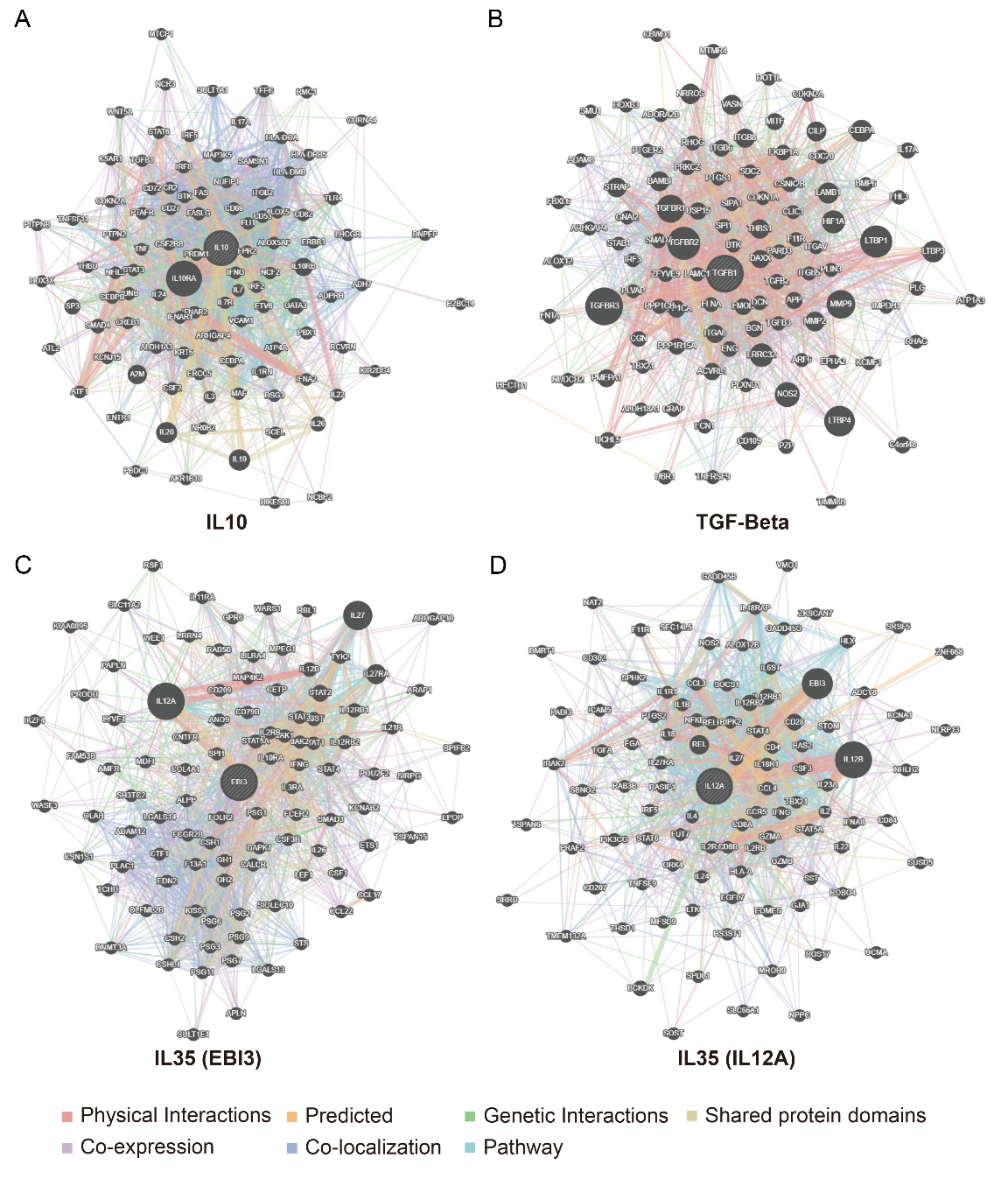


Fig. S3 Top 100 co-expression genes associated with IL10 (**A**), TGFβ (**B**), EBI3 (**C**), and IL12A (**D**). The IL-35 protein is encoded by the genes EBI3 (Epstein-Barr virus-induced gene 3) and IL12A (interleukin 12A).


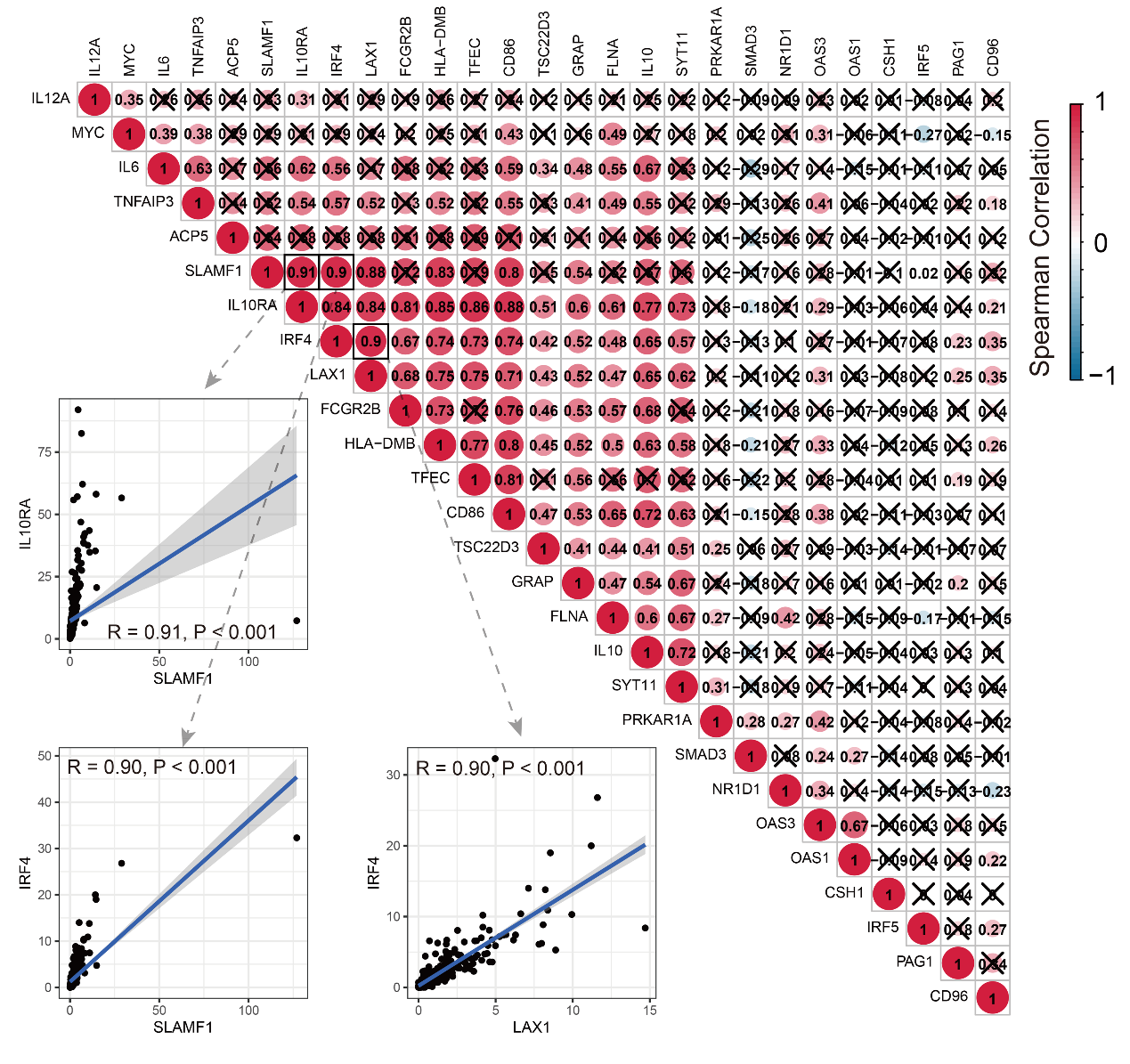


Fig. S4 Spearman correlation analysis of the 27 Breg-related genes in the TCGA-BLCA cohort.


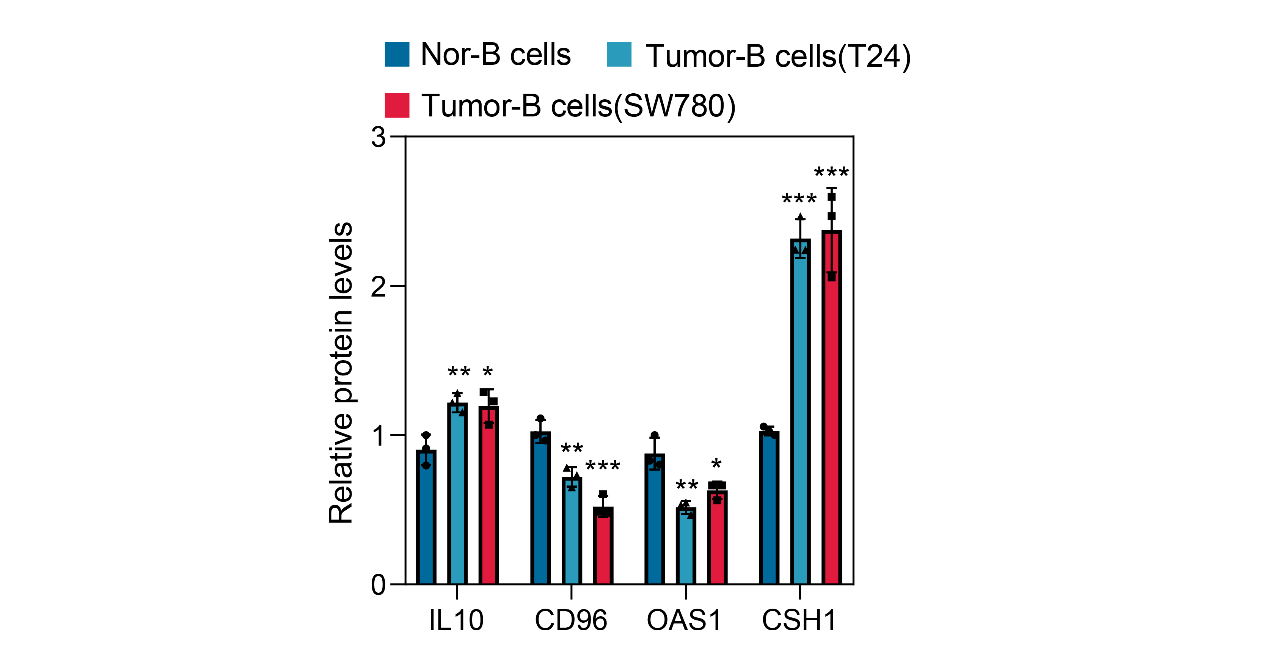


Fig. S5 The protein expressions of IL10, CD96, OAS1, CSH1 in the B cells treated with or without T24 and SW780 cells. **P < 0.05; **P < 0.01; ***P < 0.001.*


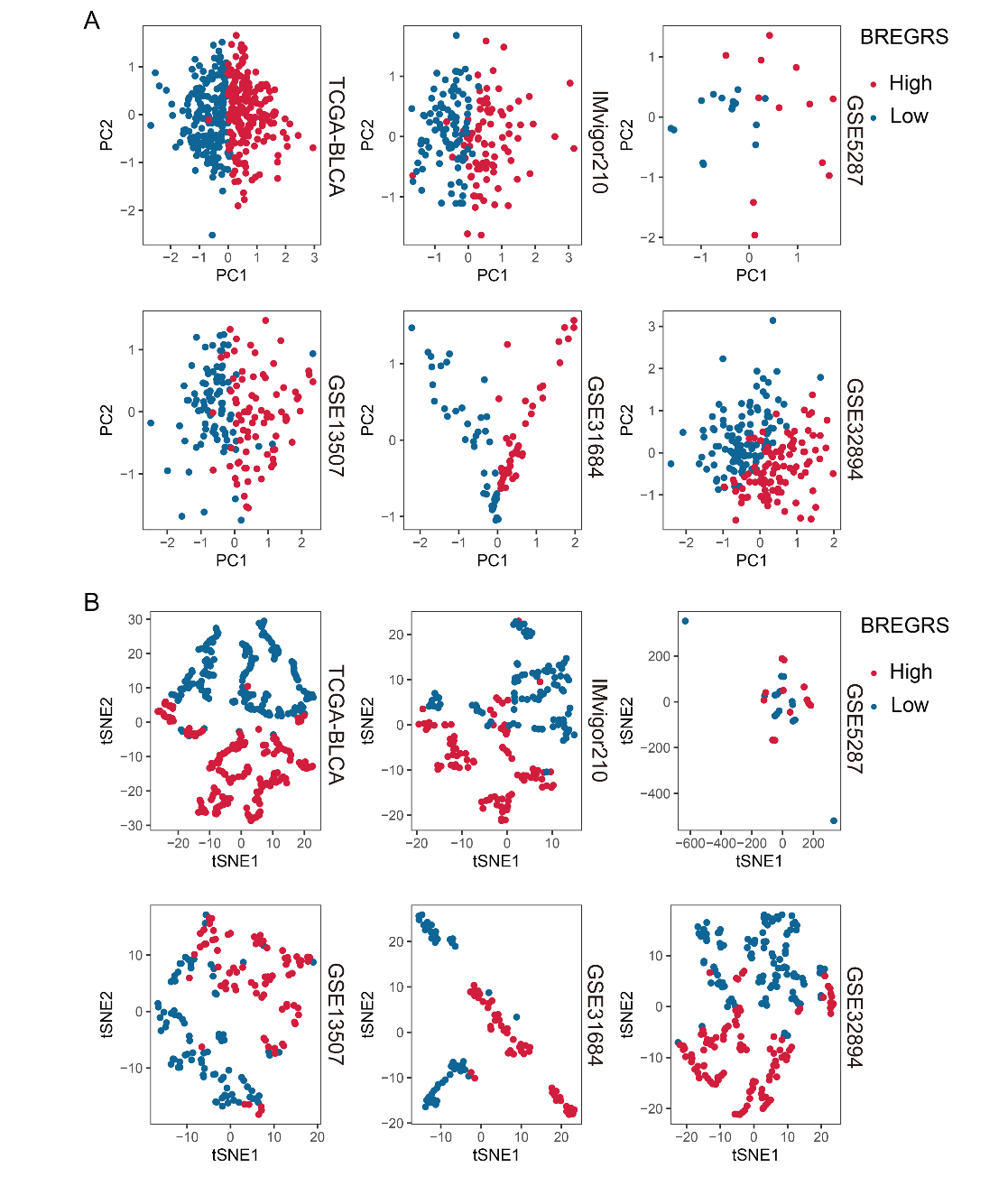


Fig. S6 Unsupervised clustering analysis using PCA (**A**) and t-SNE (**B**) reveals distinct genomic features between high- and low-BREGRS cases in the TCGA-BLCA, IMvigor210, GSE5287, GSE13507, GSE31684, and GSE32894 cohorts. *PCA, Principal Component Analysis; t-SNE, t-distributed Stochastic Neighbor Embedding; TCGA, The Cancer Genome Atlas; BLCA, bladder cancer.*


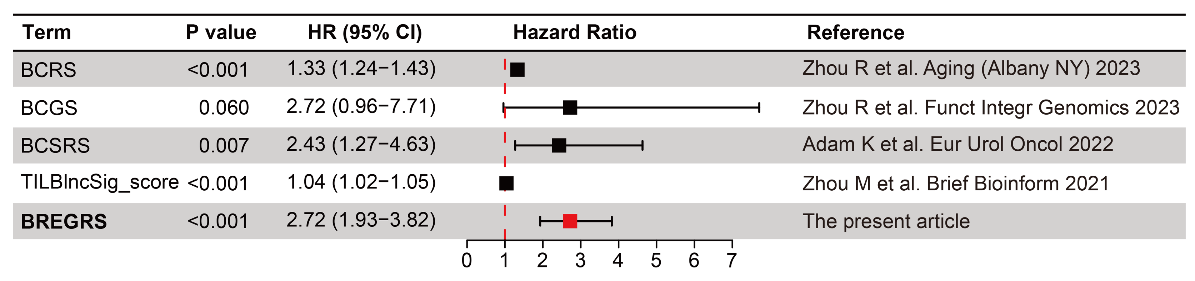


Fig. S7 Univariate Cox regression analysis demonstrates that BREGRS shows superior predictive ability for OS in the TCGA-BLCA cohort, surpassing established multiple-gene signatures associated with B cells' profiles. *OS, Overall Survival; BREGRS, Breg-related score.*


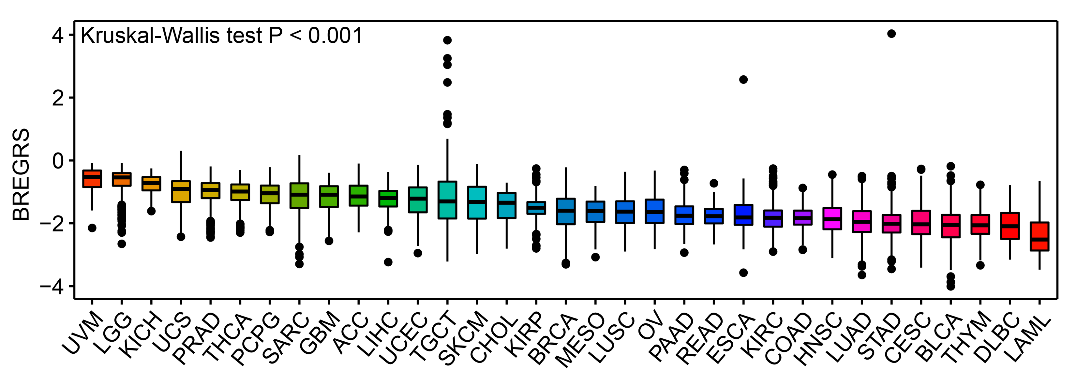


Fig. S8 Analysis of the TCGA database reveals the levels of BREGRS across 33 different cancer types.


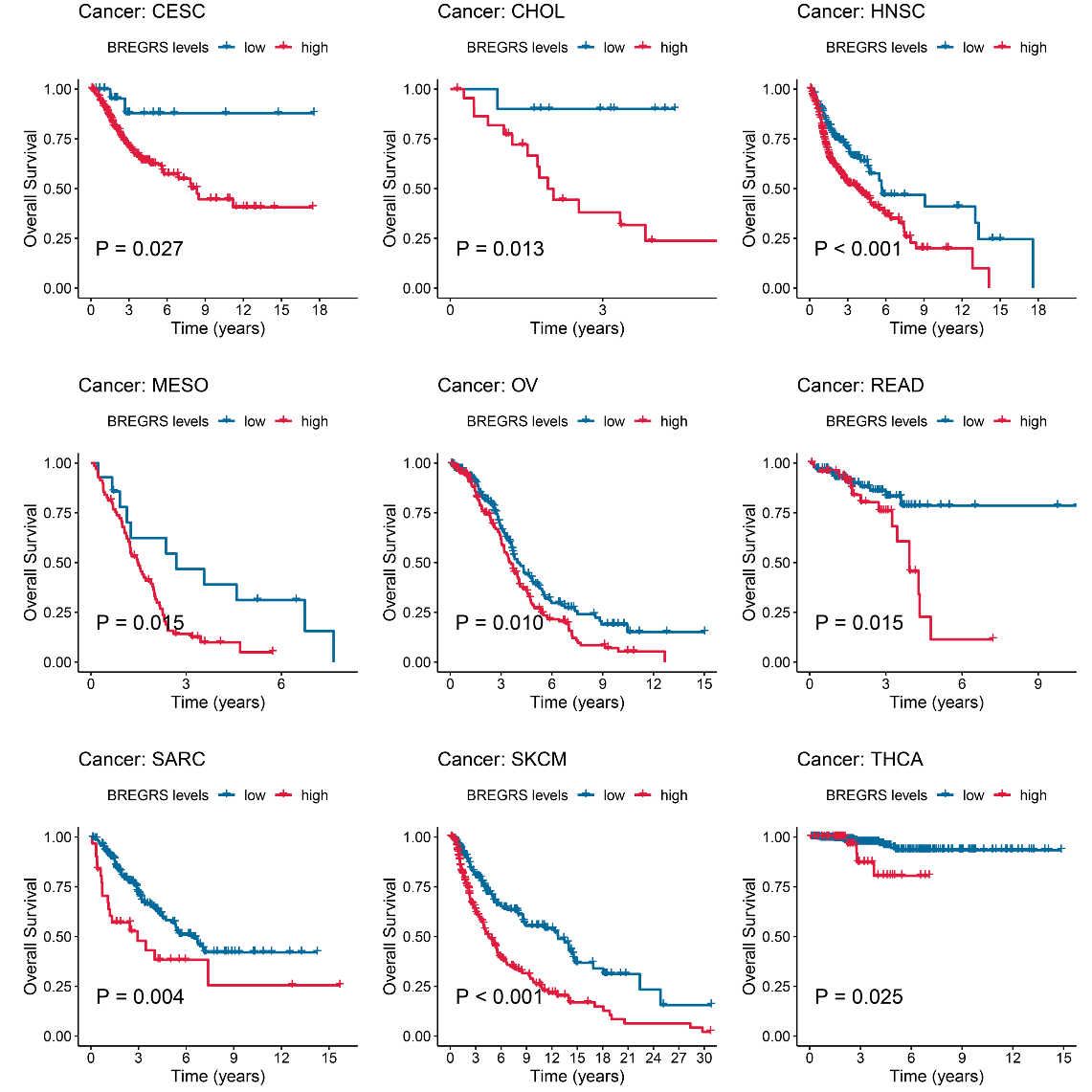


Fig. S9 High levels of BREGRS are associated with an unfavorable prognosis in 9 different cancer types. *CESC, cervical squamous cell carcinoma and endocervical adeno carcinoma; CHOL, cholangio carcinoma; HNSC, head and neck squamous cell carcinoma; MESO, mesothelioma; OV, ovarian serous cystadenocarcinoma; READ, rectum adenocarcinoma; SARC, sarcoma; SKCM, skin cutaneous melanoma; THCA, thyroid carcinoma.*


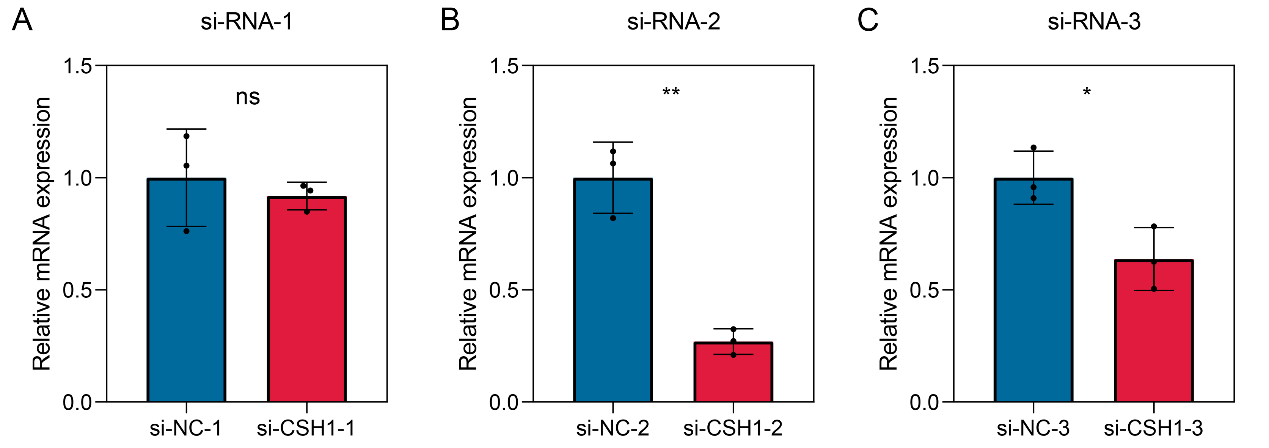


Fig. S10 The suppressive efficacy of siRNA-1 (**A**), siRNA-2 (**B**), and siRNA-3 (**C**) against CSH1, as detected by RT-qPCR experiments. *RT-qPCR, real-time quantitative PCR; ns, not significant; *P < 0.05; **P < 0.01.*


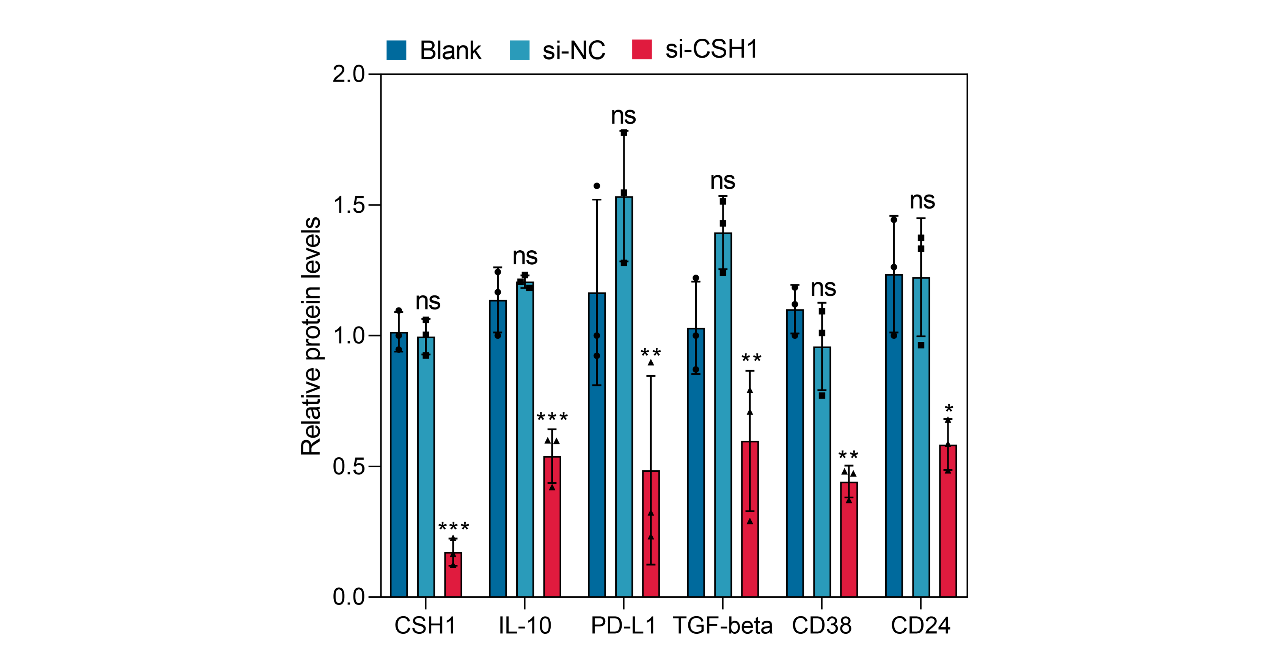


Fig. S11 The protein expressions of CSH1, IL10, PD-L1, TGFβ, CD38, and CD24 in the B cells with or without CSH1 knockdown. **P < 0.05; **P < 0.01; ***P < 0.001.*


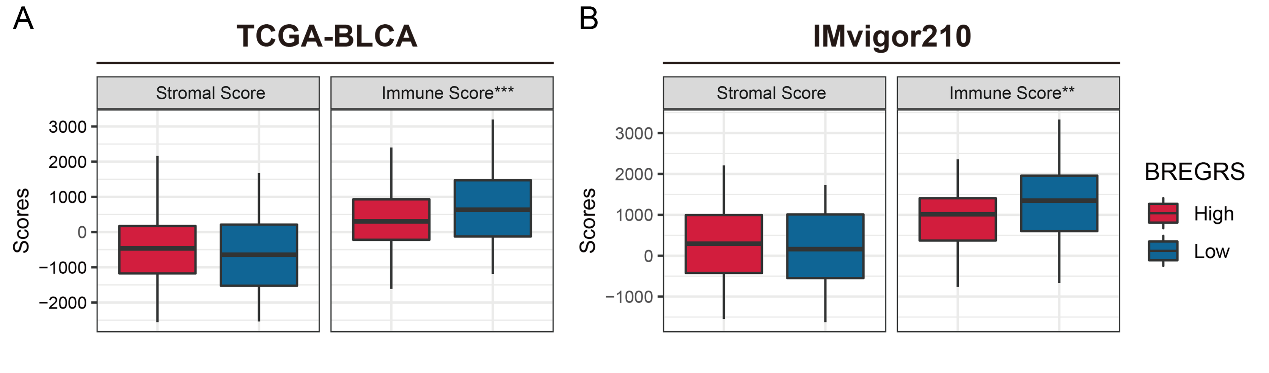


Fig. S12 The high-BREGRS subjects exhibit lower immune components of the tumor microenvironment, both in the TCGA-BLCA (**A**) and IMvigor210 (**B**) cohorts. ***P < 0.01; ***P < 0.001.*


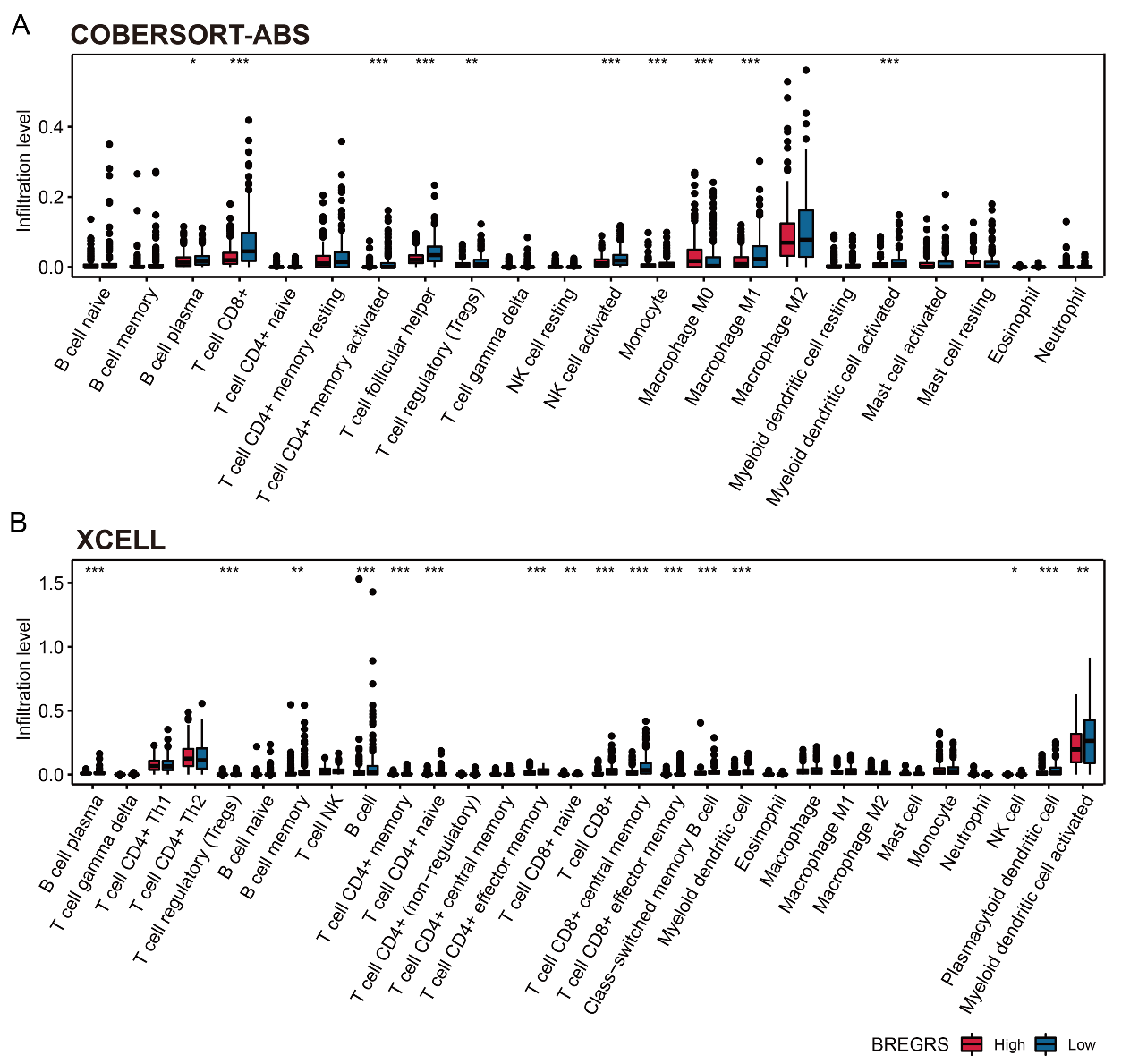


Fig. S13 The infiltration levels of immune cells in the tumor microenvironment of TCGA-BLCA subjects based on the CIBERSORT-ABS (**A**) and XCELL (**B**) algorithms. **P < 0.05; **P < 0.01; ***P < 0.001.*


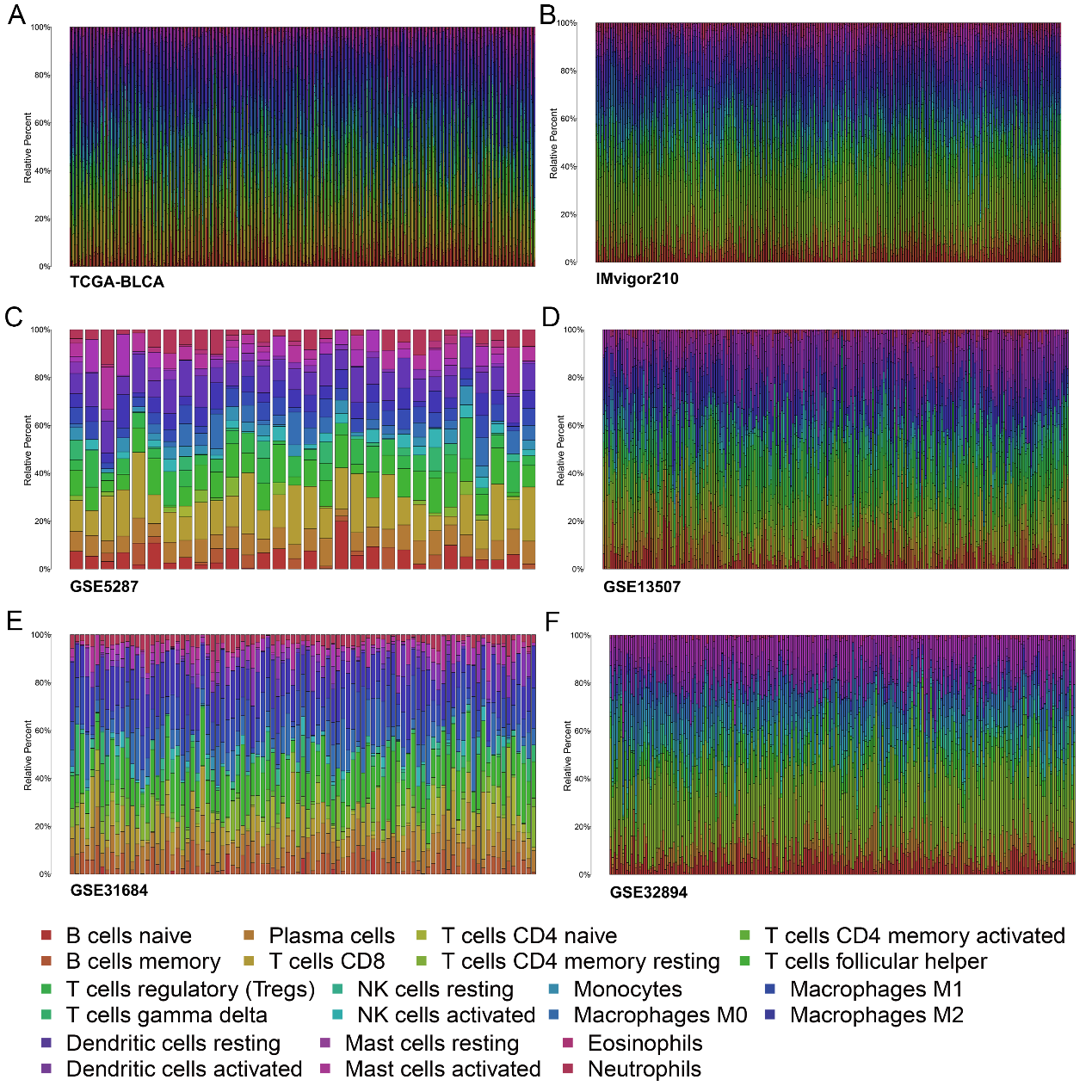


Fig. S14 The CIBERSORT-ABS algorithm was used to evaluate the infiltration proportion of immune cells in the TCGA-BLCA (**A**), IMvigor210 (**B**), GSE5287 (**C**), GSE13507 (**D**), GSE31684 (**E**), and GSE32894 (**F**) cohorts using the “immunedeconv” package of R.


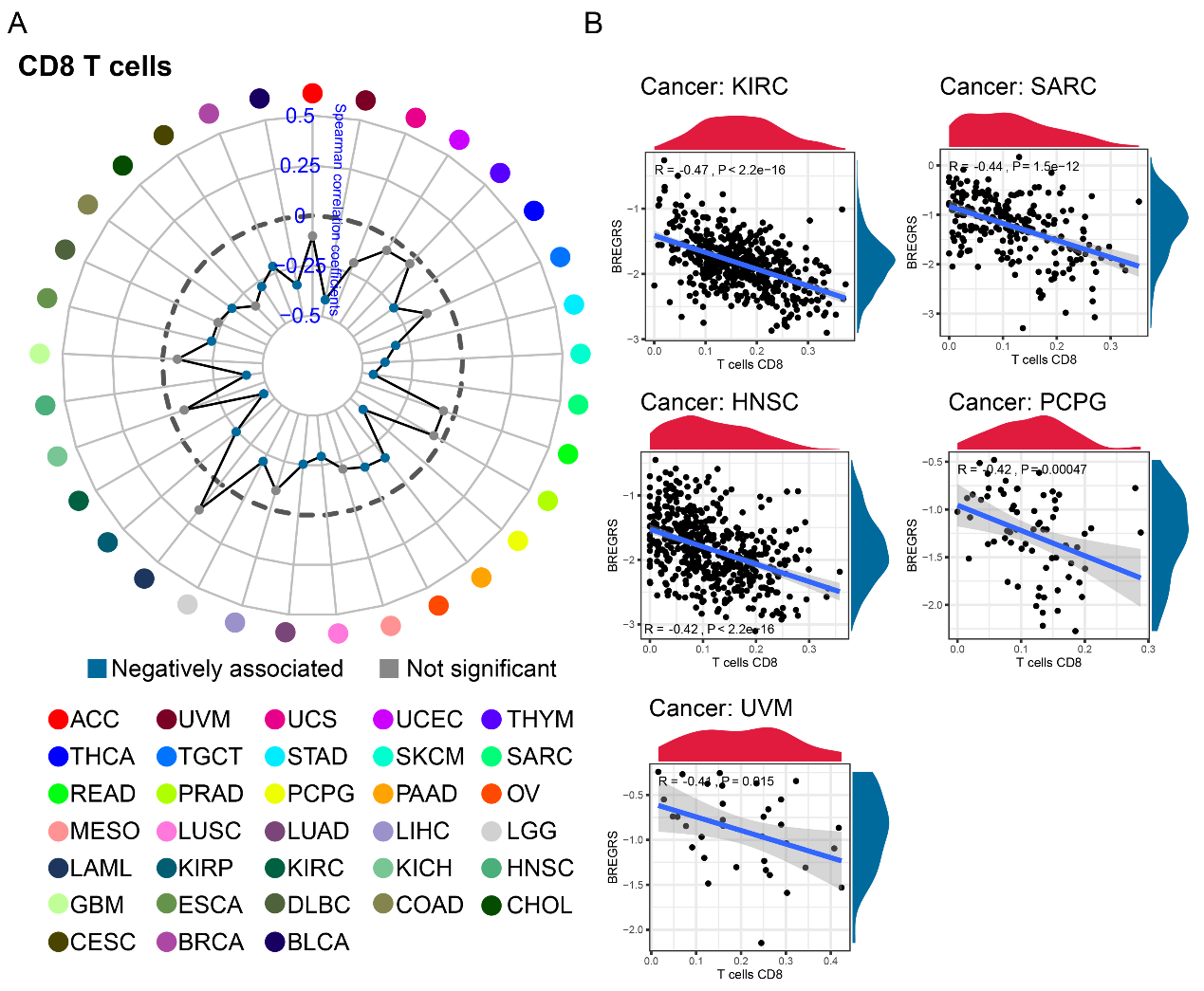


Fig. S15 Correlation between BREGRS and CD8+ T cells' infiltration proportion in pan-cancer analysis. The infiltration levels of CD8+ T cells were evaluated using the CIBERSORT-ABS algorithm in subjects from TCGA. (**A)** BREGRS demonstrated a negative association with CD8+ T cells' infiltration proportion in the majority of cancers. (**B)** The top 5 cancers displayed the strongest correlation coefficients between BREGRS and CD8+ T cells' levels. *KIRC, kidney renal clear cell carcinoma; PCPG, pheochromocytoma and paraganglioma; UVM, uveal melanoma.*


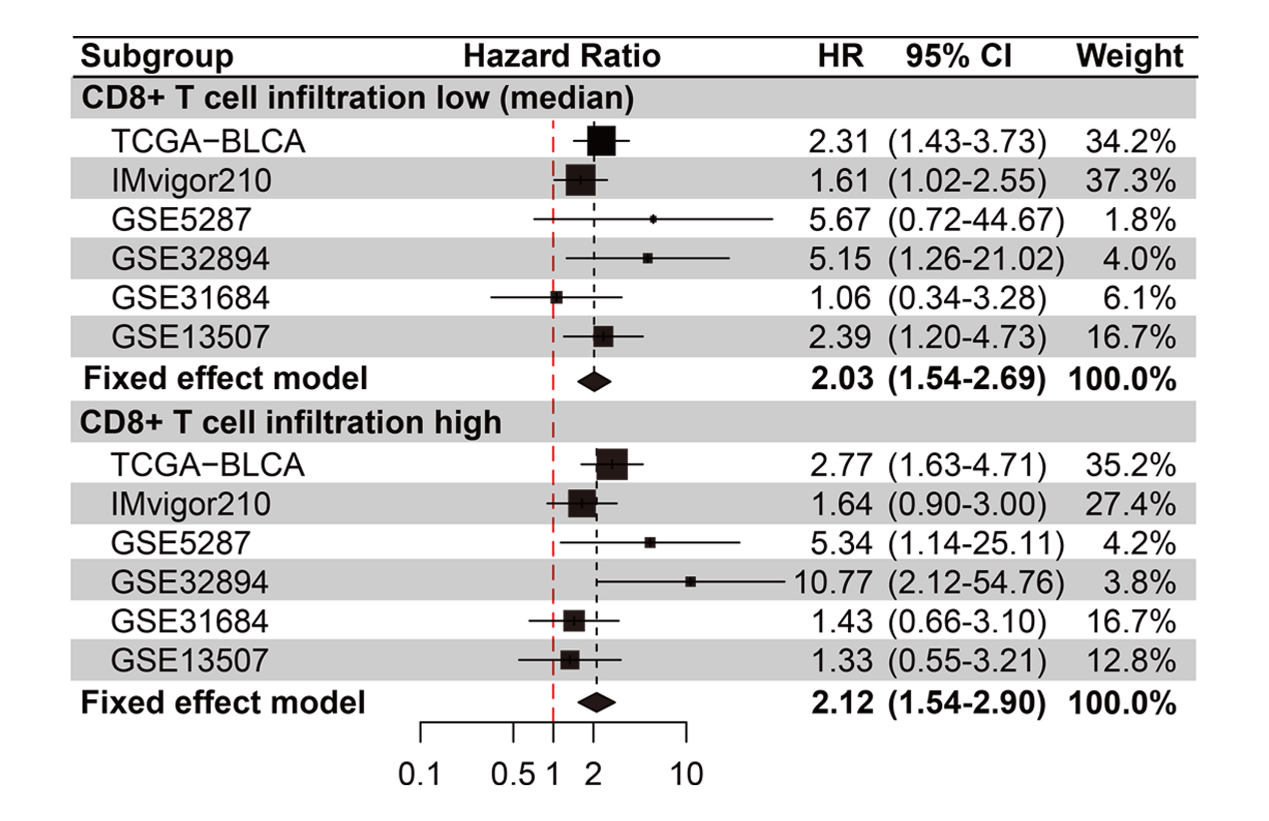


Fig. S16 BREGRS served as a significant prognostic indicator in both low- and high-CD8+ T cell infiltration levels.


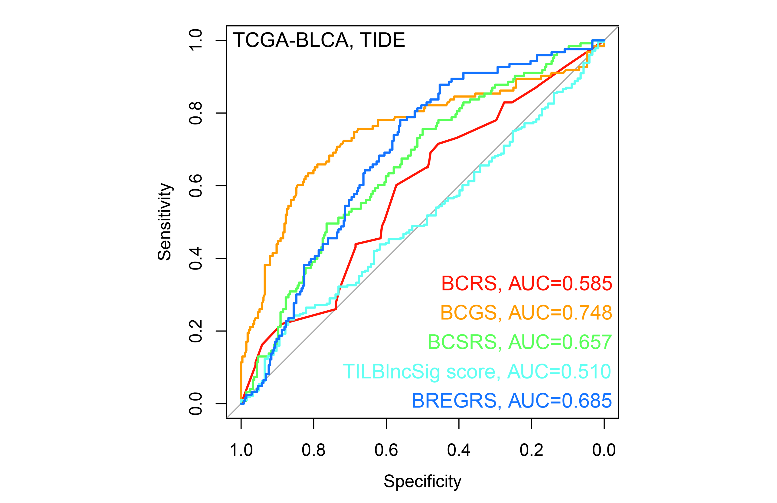


Fig. S17 Predictive ability of BREGRS and established multiple-gene signatures associated with B cells' profiles in the immunotherapeutic response of the TCGA-BLCA cohort. The immunotherapeutic response of subjects from the TCGA-BLCA cohort was assessed using the TIDE algorithm. *TIDE, Tumor Immune Dysfunction and Exclusion; AUC, Area Under Curve.*


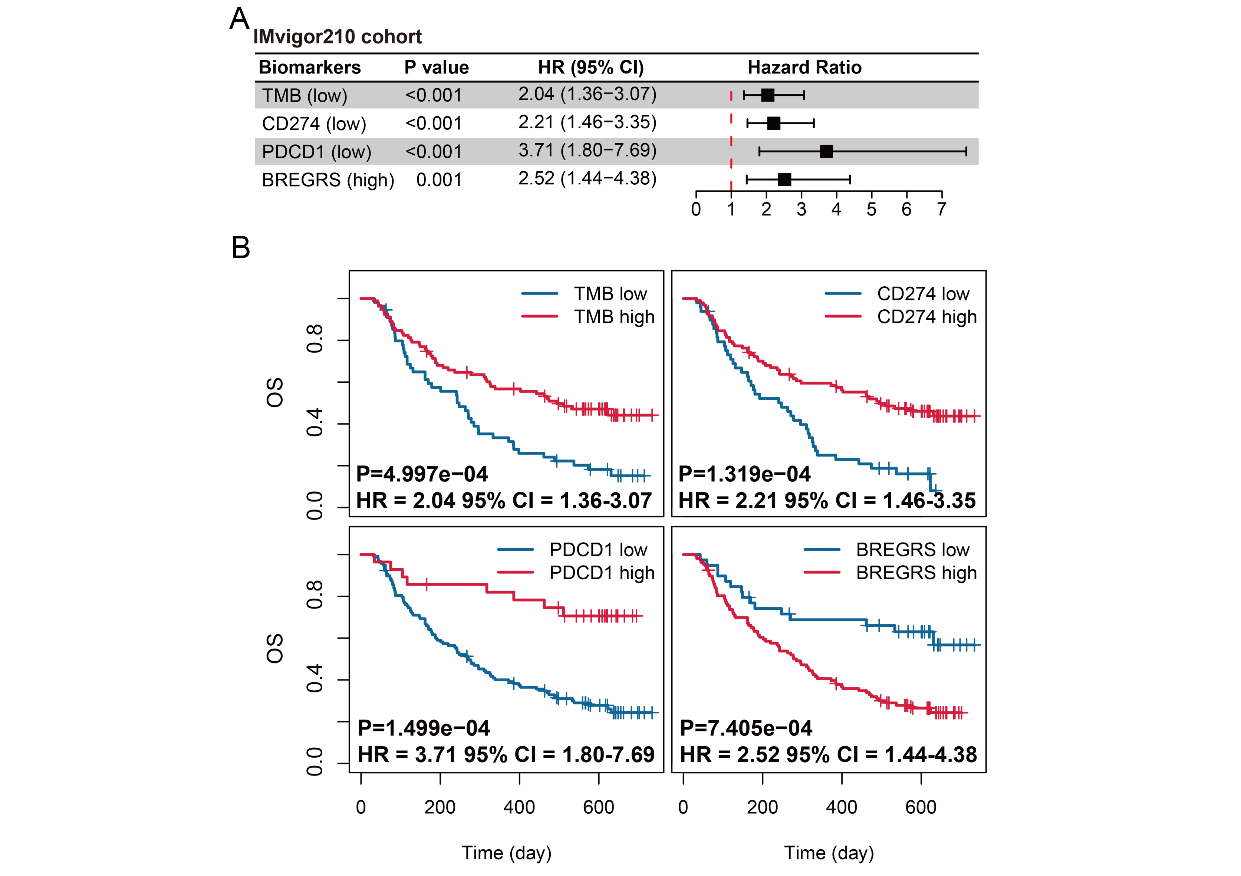


Fig. S18 Comparison of BREGRS and widely-accepted immunotherapeutic biomarkers for predicting OS in subjects from the IMvigor210 cohort. (**A)** Univariate Cox regression analysis was performed to assess the predictive ability of BREGRS and established immunotherapeutic biomarkers for OS. (**B)** Kaplan-Meier log-rank tests were used to compare the OS outcomes between different groups based on BREGRS and the immunotherapeutic biomarkers.
